# Recent advances in the medical applications of hemostatic materials

**DOI:** 10.7150/thno.79639

**Published:** 2023-01-01

**Authors:** Yu Guo, Min Wang, Qi Liu, Guoliang Liu, Shuang Wang, Jiannan Li

**Affiliations:** 1 Department of the General Surgery, Jilin University Second Hospital, Changchun, China.; 2 Department of Operating Theater and Anesthesiology, Jilin University Second Hospital, Changchun, China.; 3 Department of the Dermatology, Jilin University Second Hospital, Changchun, China.

**Keywords:** hemostatic materials, polymer, nanofibers, hydrogels, medical use.

## Abstract

Bleeding caused by trauma or surgery is a serious health problem, and uncontrollable bleeding can result in death. Therefore, developing safe, effective, and convenient hemostatic materials is important. Active hemostatic agents currently used to investigate the field of hemostasis are divided into four broad categories: natural polymers, synthetic polymers, inorganic materials, and metal-containing materials. Hemostatic materials are prepared in various forms for wound care applications based on the active ingredients used. These materials include nanofibers, gels, sponges, and nanoparticles. Hemostatic materials find their applications in the field of wound care, and they are also used for hemostasis during malignant tumor surgery. Prompt and effective hemostasis can reduce the possibility of the spread of tumor cells with blood. This review discusses the outcomes of current research conducted in the field and the problems persisting in the field of developing hemostatic materials. The review also presents a platform for the further development of hemostatic materials. Bleeding caused by trauma or surgery is a serious health problem, and uncontrollable bleeding can result in death. Therefore, developing safe, effective, and convenient hemostatic materials is important. Active hemostatic agents currently used to investigate the field of hemostasis are divided into four broad categories: natural polymers, synthetic polymers, inorganic materials, and metal-containing materials. Hemostatic materials are prepared in various forms for wound care applications based on the active ingredients used. These materials include nanofibers, gels, sponges, and nanoparticles. Hemostatic materials find their applications in the field of wound care, and they are also used for hemostasis during malignant tumor surgery. Prompt and effective hemostasis can reduce the possibility of the spread of tumor cells with blood. This review discusses the outcomes of current research conducted in the field and the problems persisting in the field of developing hemostatic materials. The review also presents a platform for the further development of hemostatic materials.

## Introduction

Post-traumatic hemorrhage is the second leading cause of human trauma-related deaths, accounting for 15% of all trauma deaths 1, 2. The amount of blood in a normal person remains relatively constant during the life of the person, and the blood volume is proportional to the person's body weight. The amount of blood in a normal human has been reported to be approximately 7-8% of the total body weight 3. When blood loss reaches 20% of total blood volume, it becomes difficult to maintain normal blood volume and blood pressure. Symptoms such as dizziness and reduced urine output are observed under these conditions. When the extent of blood loss exceeds 40% in a short time, life-threatening conditions develop if blood is not transfused in time 4. Therefore, it is important to control the extent of bleeding. Cessation of bleeding from small wounds is realized through a series of events such as vasoconstriction, platelet thrombosis, and blood clotting 5.

The rupture of blood vessels can result in vasoconstriction, reducing blood flow to the injured site. At the same time, some hemostatic agents, such as thromboxane, are released into the bloodstream. These promote the local constriction of small arteries 6. Injury exposes subendothelial collagen and a small number of platelets adhering to the damaged endothelium. The release of adenosine diphosphate (ADP) from erythrocytes and thrombin generated during coagulation can trigger the induction of the functions of platelets. The activated platelets promote the release of endogenous ADP and thromboxane 2 (TXA2), resulting in the activation and recruitment of a large number of platelets. This results in the formation of platelet thrombi that plug wounds, and the process is known as primary hemostasis 7. The process of coagulation cascade can activate multiple coagulation factors in a certain sequence following two pathways (endogenous and exogenous). Eventually, soluble fibrinogen in plasma is converted into insoluble fibrin, which interweaves to reinforce the hemostatic plug. The endogenous coagulation pathway is initiated by the activation of a surface factor (Factor XII) by damaged endothelial cells, whereas the exogenous coagulation pathway is activated following the exposure of a tissue factor (Factor III) to blood. The two pathways converge by activating the respective downstream coagulation factors. This results in the activation of the Stuart Prower factor (Factor X) pathway, which activates thrombinogen. Thrombin could activate the fibrin stabilizing factor (Factor XIII), which, in the presence of Ca^2+^, could cause the fibrin monomers to polymerize with each other, resulting in the formation of water-insoluble multimeric clots. The platelet emboli formed during the primary hemostasis phase are reinforced under these conditions 8, 9. Under normal circumstances, rapid hemostasis can be achieved in the presence of a large number of local adherent platelets and clotting factors. Thrombotic disorders are observed when the number of platelets increases beyond a certain range 10. However, severe post-traumatic bleeding requires aggressive human intervention to promote coagulation. Uncontrolled post-traumatic hemorrhage is one of the leading causes of preventable death. Massive blood loss can sometimes lead to unavoidable serious complications requiring amputation. Severe infection may also occur 11.

It is important to develop safe, efficient, and stable hemostatic materials urgently. Hemostatic materials can be used to stop bleeding, and the process is realized following various ways. For example, gauze, a traditional hemostatic material, can be used to stop bleeding under conditions of physical compression (realized using pressure wraps) 12. Hemostatic sponges function as efficient absorbents. These swell when local blood gets absorbed, resulting in local compression. Polyurethane (PU) sponges, which are used for local compression, are used as nasal tamponades 13. Several hemostatic materials can activate the hemostatic pathways in the human body in various ways. For example, chitosan sponges can promote hemostasis by activating and aggregating platelets exploiting the negative charges on the chitosan surface 14. At the same time, the absorbent property of the sponge helps increase the local concentration of platelets and coagulation factors in the wound. This results in the promotion of the coagulation cascade. Several hemostatic materials can also be loaded with hemostatic drugs. It has been observed that the thrombin-loaded gelatin sponges that have been used to study a porcine liver hemorrhage model can be used to achieve excellent hemostasis 15. Nanofibers have been found to be excellent drug carriers, and these can be used to stop bleeding and promote the process of wound healing 16, 17. There are many commercially available hemostatic materials, such as Hemcon® (chitosan-based), TraumaDEX® (Potato starch-based microparticles), and QuickClot® (zeolite-based), which can efficiently reduce the bleeding time to a certain extent. These agents exhibit antimicrobial activity but also suffer from poor biodegradability. These cannot be used to treat complex wounds and are difficult to remove.

Natural polymers, synthetic polymers, inorganic materials, and metal-containing materials are primarily used to realize hemostasis (Table 1). Natural polymers used in the field of medicine for hemostasis include chitosan, cellulose, hyaluronic acid (HA), alginate, collagen, and fibrin. Peptides, polyethylene glycol (PEG), polyurethane (PU), polyvinyl alcohol (PVA), polycaprolactone (PCL), and poly (ethylene oxide) (PEO) are used to prepare the synthetic hemostatic materials. Most studies on inorganic hemostatic materials have focused on zeolites, mesoporous silica, and graphene. Calcium, zinc, and silver are primarily used to prepare metal-containing hemostatic materials. It is challenging to process these materials containing different types of active ingredients and develop different chemically modified hemostatic materials that exhibit powerful wound healing properties in addition to the basic hemostatic properties. The type of hemostatic material is strongly related to its function. Nanocellulose, hydrogels, nanoparticles, *etc.*, are widely used for the development of hemostatic materials. Chitosan hydrogels, chitosan nanoparticles, and chitosan hemostatic sponges, which can be used efficiently to treat a wide range of wounds, are different types of chitosan-based hemostatic materials. Hemostatic materials in the form of hydrogels tend to promote clotting by thickening the blood and increasing the concentration of platelets and clotting factors. These materials exert a local compression effect by expanding in size after absorbing water. However, hydrogels are notoriously difficult to remove and cannot be efficiently used to treat complex, deep wounds. Nanoparticles can be used effectively to realize the hemostasis of deep and irregular wounds. However, these are difficult to remove. Different forms of hemostatic materials prepared using the same active ingredient can be used to treat different types of wounds. For example, Hemcon® and Chitoflex® are lyophilized chitosan-based materials used to treat post-traumatic or surgical bleeding. It should be noted that Chitoflex® is more flexible than Hemcon®, and the former is more suitable than the latter to stop bleeding occurring near joints. The composite material is strong and suitable for treating complex and diverse wounds.

This review presents the mechanisms through which different substances promote coagulation. We also present the results obtained when various nanomaterial-based hemostatic materials were studied. Finally, we have focused on the pros and cons of the diverse forms of hemostatic materials and have reported the application prospects and importance of hemostatic materials in the field of healthcare. The data presented herein provide an outlook for future research directions.

### Chemical composition of hemostatic materials

Natural polymers, synthetic polymers, inorganic materials, and metal-containing materials are currently used to prepare hemostatic materials. In this section, we present the mechanisms following which these substances accelerate wound hemostasis.

#### Natural polymers

The widely available natural polymeric hemostatic materials currently in use are polysaccharides and collagens. Polysaccharides exhibit excellent properties. These are non-toxic to human tissues. These are non-irritants, non-immunogenic, non-hemolytic, histocompatible, and naturally degradable. These materials are widely used to manufacture artificial skin 45, 46, absorbable sutures 47, 48, and drug carriers 49-51. Well-studied polysaccharide-based hemostatic materials include chitosan, cellulose, HA, and alginate.

Chitosan is a natural polysaccharide extracted from the shells of crustaceans. Chitosan can increase the intracellular calcium ion concentration of platelets, facilitating platelet aggregation and mutual adhesion 52. Activation of platelets results in the production of a large number of negatively charged phosphatidylserine units on the surface, which can interact electrostatically with positively charged chitosan, increasing the viscosity of platelets. This results in the rapid formation of thrombi. The positive charge on the surface of chitosan polymers interacts with receptors containing neuraminic acid residues (negative charge) on the surface of red blood cells to promote aggregation 53. Chitosan is abundant in nature and can be modified to improve hemostatic effects and increase the application prospects of chitosan-based materials. The cationic properties of chitosan significantly influence the function of the material as a coagulant of red blood cells and platelets. Tan* et al.* reacted *N,N,N*-trimethyl chitosan with chloroacetyl chloride to obtain chitosan derivatives. Subsequently, they reacted tricyclohexylphosphine with triphenylphosphine to obtain highly cationic chitosan derivatives 54. The introduction of quaternary ammonium groups into chitosan improved the blood cell adhesion and antibacterial properties of the material 55. Wibel* et al.* formulated thiol chitosan by incorporating L-cysteine with amide bonds. This helped improve the mucosal adhesion and penetration ability of the materials 56. Jayakumar* et al.* synthesized phosphorylated chitosan by grafting mono(2-methacryloyloxyethyl) ester of phosphoric acid into chitosan to improve the stability and antimicrobial properties of the materials 57. In addition to direct modification, Schnurch* et al.* grafted ethylenediaminetetraacetic acid (EDTA) onto chitosan polymers. The chelation of magnesium ions resulted in the generation of antimicrobial effects 58. Alkylated chitosan units are hydrophobic, and these can be inserted into blood cell membranes to promote blood clotting 59. The hydrophobically modified surface hinders the process of local fluid exchange between wounds and the external environment. This helps prevent bacterial and other contamination of the wounds. Chitosan-based hemostatic materials have been extensively studied in rat wound models, rat femoral artery trauma models, and rat liver injury models.

Commercially available chitosan-based hemostatic materials (*e.g.,* Hemcon®, Chitoflex®, and CELOX®) have exhibited excellent efficacy in trauma and surgical hemostatic applications. Chitosan-based hemostatic materials exhibit limited hemostatic effects and cannot be used to effectively control extensive bleeding 60. These limit the application prospects of these materials.

Cellulose is a macromolecular polysaccharide that is present in abundance in plants and bacteria. It is used for hemostasis and can be used to absorb water quickly. Cellulose fills the site of bleeding, providing physical compression to stop bleeding 61. Cellulose contains acidic carboxyl functional groups, which can bind with Fe^3+^ ions in hemoglobin. This results in the formation of brown gels that help close capillary ends and stop bleeding 62. It can potentially activate platelets to accelerate hemostasis 63. Oxidized cellulose (OC) is a cellulose derivative that is synthesized following the oxidation of TEMPO (2,2,6,6-tetramethyl-1-piperidinyloxy). The method helps increase the anionic carboxyl group content on the cellulose surface. A large number of hydroxyl groups are oxidized to carboxyl groups to reduce surface water repellency 64, 65. OC introduces a large number of carboxyl groups on the surface, lowering the local pH and causing non-specific platelet aggregation. This can potentially result in thrombosis and the promotion of hemostasis. The process can help in wound healing. The relatively low pH of OC helps inhibit the growth of a wide range of gram-positive and gram-negative microorganisms 66. Cellulose is commonly prepared as hemostatic material together with other active coagulation ingredients. For example, OC-based nanofibers loaded with thrombin exhibit excellent biocompatibility and rapid water absorption properties. These can be used to study models of rat liver injury to significantly shorten clotting time and reduce bleeding volume 67. The OC-based commercial hemostatic material Surgicel^®^ is particularly effective in stopping bleeding during surgery. It can also be used to stop small arterial bleeding. The material is used during neurosurgery to reduce intracranial hematomas in bleeding patients 33. However, the acidic environment can potentially generate an inflammatory response in tissues, prolonging the wound-healing process. This may also damage peripheral nerves. The slow degradation of cellulose-based hemostatic materials significantly hinders the application prospects of the materials. These may cause foreign body reactions and adhere to the wound area, interfering with epithelial growth and resulting in delayed healing 68, 69.

HA, an acidic mucopolysaccharide, is a natural component of the body with excellent biocompatibility 70. HA contains a large number of hydroxyl groups and can form hydrogen bonds with water molecules to absorb water and increase the concentration of platelets and clotting factors 71. It is also involved with the process of releasing inflammatory cytokines (such as TNF-α, IL-1β, and IL-8) 72. It directs the processes of fibroblast invasion and proliferation to promote wound healing 71, 73. It should be noted that HA is easily soluble in water and has a short residence time in tissues. The mechanical properties of HA were improved, and its degradation rate was controlled following the processes of cross-linking 74, 75, esterification 76, 77, molecular modifications 78, 79, and compounding 80, 81. The addition of a certain amount of hydrazinolyzed 3,3'-dithiopropionic acid (DTP) to an aqueous solution of HA to obtain a mercapto-hyaluronic acid derivative (HA-DTPH) resulted in a decrease in the rate of degradation of HA and an improvement in the wet tissue 82, 83. Some researchers used tyramine to modify HA by introducing phenol groups to the backbone of HA. This resulted in the production of hydrogels with controlled mechanical strength 84. Hydrogels based on HA-tyrosine (HA-Tyr) prepared following the process of enzymatic cross-linking could improve the extent of hemostasis achieved and effectively increase the cell adhesion property of the material 74, 85-87. Bagheri* et al.* studied the efficacy of phenolic hydroxy HA (HA-Ph)-based hydrogels (characterized by different degrees of cross-linking) on the cell adhesion and proliferation properties of the materials 75. The drawbacks of using modified HA as a hemostatic material lie in the difficulty of removing the material when necessary. HA-based hemostatic materials have been examined in the mouse model of total skin injury, the arterial hemorrhage model, the liver injury model, and the porcine left ventricular penetration model 24.

Alginate, similar to HA, is highly absorbent and can increase the concentration of platelets and coagulation factors. Alginate can increase the local Ca^2+^ concentration and accelerate the coagulation process 88, 89. Furthermore, alginate exhibits cellular chemotactic activity, which promotes wound repair. Alginate oligomers (AOS), which retain the physical and chemical properties of the parent polymer and can be modified, are the focus of current research 90, 91. The hydroxyl group in the glyoxylate units can be transformed into an aldehyde group in the presence of strong oxidizing agents. This transformation helps to improve the degradation properties of the material. AOS can cross-link with various metal ions, such as Ca^2+^, to form gels that improve hemostasis and viscosity 92-95. Alginate is not designed to treat dry wounds with hard crusts. Numerous studies have been conducted on the diabetic foot ulcer model, and the alginate-based commercial product Algosteril^®^ has been approved clinically for wound care dressing.

Collagen, a porous material, expands following blood absorption. It forms a mesh for blood clotting and sealing cracks in blood vessels or wounds. It can activate platelets and induce the release of physiological aggregating agents such as ADP and TXA2, resulting in the irreversible aggregation of platelets. It also functions as a platelet adhesion matrix that helps form blood clots for hemostasis 96-98. Collagen promotes the process of proliferation during the wound repair process 99. Collagen tends to cause allergic reactions, exhibits poor adhesion properties, and cannot be easily degraded, and these properties hinder the practical applications of the material. Partial hydrolysis of collagen results in the production of gelatin, and it can be used to address (to a certain extent) the safety issues posed by collagen 100. Gelatin has been processed in various forms for clinical applications, and the most commonly used gelatin-based material for hemostasis is the gelatin sponge 101, 102. Slezak* et al.* compared the hemostatic effects exerted by the collagen and thrombin-containing powder agent Hemoblast® and the flowable gelatin-thrombin matrix Floseal® in a porcine liver hemorrhage model. The results revealed that the gelatin-the thrombin-based system was more efficient than the other model in reducing bleeding. The ease of applicability of the former was better than that of the latter 15. Wang* et al.* used gold/silver clusters to functionalize gelatin sponges that exhibited good antibacterial properties 103. Additionally, gelatin and collagen are popular materials for wound healing because they are biocompatible and biodegradable. Commercial gelatin-based hemostatic products, such as Floseal® and Endo Avitene®, exhibit excellent hemostatic efficacy in clinical settings. FloSeal**^®^** is a commercial hemostatic material consisting of animal collagen, glutaraldehyde, and human coagulation enzymes in fibrin glue. It is currently used to treat obstetric bleeding 104, nasal bleeding 105, 106, and joint bleeding 107. Moreover, it is also used for intracranial tumor removal 108. Collagen and gelatin are prone to allergic reactions and are liable to degradation.

Fibrin significantly affects the coagulation process and can form insoluble fibrin multimers under the effect of thrombin and Ca^2+^. Multimers trap platelets and blood cells to form blood clots 109, 110. It can also stimulate the growth of capillary endothelial cells and fibroblasts, and the process promotes wound healing 111. Tissuccol^®^, a commercial fibrin glue, produced satisfactory hemostatic results when used to treat upper gastrointestinal bleeding and splenic rupture. It could also be used to treat hepatectomy 81 efficiently. Fibrin-based materials cannot be used to stop bleeding from large arteries and veins, and this can be attributed to the long clotting time. The use of fibrin-based materials may result in animal or human blood-borne infections 112.

Natural polymers can facilitate platelet aggregation and activation through various mechanisms to achieve rapid hemostasis and promote wound healing. The aldehyde group in oxidized dextran can serve as a cross-linking agent for the preparation of hydrogels and sponges, and it can also cross-link with amino groups on the tissue surface to promote the local adhesion of hemostatic materials 113. The high price, poor mechanical properties, and tendency of natural polymers to cause allergic reactions limit the development of natural polymer-based hemostatic materials.

#### Synthetic polymers

It is easier to functionalize synthetic polymers synthesized following physical or chemical methods than natural polymers. Hence, these materials have attracted immense attention in the field of research of hemostatic material research.

Solutions made of self-assembling peptides can gelate rapidly with blood, and the formed hydrogel can seal wounds. Thus, such hydrogels can be used as hemostatic agents. The molecules in the peptide solution can trigger the processes of self-assembly and gelation when they encounter charged amino acid molecules in the blood. When applied to a wound as hemostatic material, the peptide can rapidly absorb the surrounding fluid, increase the local concentration of platelets and clotting factors in the wound, initiate the peptide self-assembly process, and form a nanofibrous scaffold to promote the exudation of red blood cells and blood from wounds 114. Polylysine contains abundant primary amine groups that are cationic in neutral aqueous solutions. The ionic attraction between these amino groups and the residues exposed under conditions of tissue damage demonstrate the potential tissue adhesion property of polylysine 115. Nie *et al.* cross-linked thiolated chitosan and ε-polylysine through maleimide groups to form (*in situ*) a fast-curing hydrogel that could effectively adhere to rat liver wounds and help achieve rapid hemostasis 27. Zhang* et al.* realized the *in situ* preparation of hydrogels characterized by high mechanical strength and short gelling time by cross-linking polyacrylamide units containing a large number of amide groups with ε-polylysine. The prepared systems were used to study a model of rat liver injury, and excellent tissue adhesion and hemostatic properties were observed. It was observed that the extent of bleeding from the synthetic hydrogel-treated wounds was 1/7th of the extent of bleeding observed for the untreated groups 116. Peptide sequences other than polylysine also perform well in the field of hemostatic materials. Charbonneau* et al.* prepared a peptide sequence consisting of lysine and leucine residues bound to the surface of a biocompatible polymeric hydrogel matrix to form a hemostatic material. This material was used to test a rabbit ear bleeding model, and it was observed that the bleeding time could be reduced by 40% using this material. When a rabbit liver injury model was tested using the prepared peptide sequence, the extent of surface damage realized was less than the extent of surface damage realized using the commercial zeolite powder agent QuikClot**^®^** 117. RADA-16 (RADARADARADARADA) is a short peptide sequence consisting of arginine, aspartic acid, and alanine. This system exhibits self-assembling properties and can spontaneously assemble to form b-folded and nanofiber lattice structures under certain conditions. The composition of the structure is stable. The structure is adjustable and is characterized by high biocompatibility and non-immunogenicity. These materials mimic the porosity and structure of the extracellular matrix *in vivo*, can provide a suitable microenvironment for cell growth, promote the repair and reconstruction of damaged tissues, and promote the drug delivery process 118. Wang* et al.* used a RADA-16 self-assembled peptide system to rat spinal cord transection and liver injury models. They demonstrated that the peptide significantly reduced bleeding time and reduced bleeding volume 119. Yang* et al.* prepared a copper peptide-functionalized RADA16 system that was used to study a diabetic mouse model to effectively accelerate the processes of wound closure and tissue repair 26. Self-assembled peptide sequences can be potentially used to realize hemostasis, although some persistent disadvantages hinder their clinical applications. The peptides are not readily accessible, and some peptides need to be preheated and sonicated prior to use. The cost-inefficiency of the preparation process hinders the practical applications of the material.

PEG is widely applied in the medical field as they are characterized by excellent properties. Amphiphilic copolymers containing PEG are adsorbed on the surface of medical polymer materials to improve the biocompatibility of medical polymer materials in contact with blood 120, 121. PEG adheres well to tissues and is commonly used as a drug carrier that promotes slow drug release 122, 123. It is also used as a carrier of immobilized enzymes 124. Furthermore, PEG can crosslink with tissues and form physical barriers to inhibit bleeding. PEG-based hydrogels exhibit good water absorption properties, and these can increase the concentration of platelets and coagulation factors around wounds. These materials are suitable for the hemostatic closure of traumatic organ injuries 125. A high relative molecular weight characterizes Dobby PEG derivatives, and these can form hydrogels characterized by excellent water barrier and tissue activity. It can gradually be degraded in the body and completely excreted under conditions of *in vivo* hemostasis. The material degrades slowly and may cause foreign body reactions, delaying the process of wound healing. Wu* et al.* introduced cyclized succinate groups into the hydrogel matrix to enable the rapid degradation of the sealant to promote the degradation of PEG and reduce possible damage to tissues 28. PEG reacts with other polymers to form highly functional copolymers. PEG and acrylic acid can be formed following esterification reactions, and rapid gelation to form the hydrogel from the resulting poly (ethylene glycol) diacrylate (PEGDA) could be triggered using a photoinitiator in the presence of ultraviolet (UV) light at room temperature 126. This property makes the materials suitable for rapid wound closure 127. The property of tissue adhesion significantly affects the applicability of hemostatic materials. This property can be improved by functionalizing PEG with aldehyde groups or dopamine. Hydrogen or Schiff base bonds are formed, and amino groups are exposed in the tissue. Each coin has two sides, and the preparation of functionalized hemostatic materials poses difficulties.

PU, a synthetic polymer, exhibits good biocompatibility and degradability. It is easy to process and is used in a wide range of fields, such as trauma dressing and tissue engineering. PU foams can induce the processes of coagulation cascades and platelet aggregation 128. PU-based sponges can preserve wound moisture, promoting the process of repair of tissues based on the theory of “moist healing”. The mesh-like structure helps control the rate of water vapor transmission and resists bacterial invasion. The self-expanding PU foam provides rapid response and is characterized by rapid volume expansion. Close-fitting of the trauma surface can be realized using the material, and this can be used to study animal models of hypothermia in traumatic hemorrhagic shock (such as the porcine lethal liver hemorrhage model). It has been found that the materials can be used to rapidly and effectively reduce the amount of bleeding in cases of lethal trauma, significantly improve vital signs, ensure the perfusion of vital organs, and generate conditions for subsequent definitive hemostatic treatment 129. The commercial formulation NasoPore**^®^** (a PU-based foam) demonstrated improved hemostasis and provided good patient satisfaction in a clinical trial, as reported by Pawel* et al.* The researchers used the model for nasal caulking following endoscopic sinus surgery 13. The excellent tissue adhesion properties of PU make it suitable for application as a sealant in vascular procedures. Lisanne* et al.* used a PU-based adhesive to study a porcine model of vascular injury repair. The results revealed that the material could effectively improve the outcome of cardiovascular surgery, reduce the incidence of vascular anastomotic fistulae, and efficiently fix sutured vascular anastomoses rapidly and safely without interfering with the process of vascular wound healing 31. Huang* et al.* exploited the anti-inflammatory properties of PU nanoparticles (NPs) by combining them with gelatin. The material was implanted in the cortex of injured rats in a rat neurosurgical model, and anti-inflammatory PU NPs were released. The process significantly shortened the hemostasis time and reduced the extent of brain edema. The degree of the inflammatory response generated in the brain was also reduced, facilitating the process of tissue repair 30. PU materials degrade relatively slowly* in vivo* compared to other materials. The prolonged *in vivo* polymerization time of Tissue Glue**^®^**, a commercial PU-based formulation, hinders its application prospects.

The PVA-based hemostatic sponges, prepared following a series of processes such as cross-linking, foaming, curing, and sterilization, are characterized by non-fibrous porous structures 130. These perform well and exhibit excellent hydrophilic and liquid-absorbing properties. These also exhibit good biocompatibility and stable chemical properties. PVA swells following water absorption and can be used to realize localized compression and physical hemostasis. Therefore, these materials are commonly used for compression hemostasis of narrow cavities in humans (such as nasal 131, 132 and anal cavities). Kim* et al.* used PVA-based nanocellulose sponges to study a rabbit nasal mucosal defect model, and experiments demonstrated that PVA sponges significantly promoted the mucosal regeneration during the early stages of mucosal wound healing of mucosal wounds in noses 132. PVA does not contain pro-coagulants and relies primarily on the water-absorbing and swelling properties of the sponge material to stop bleeding. Thus, the material is characterized by a narrow application range and cannot be used to treat patients suffering from coagulation disorders.

PCL has been approved by the U.S. Food and Drug Administration (FDA) for a wide range of biomedical applications as it exhibits excellent biocompatibility and good biodegradability. PCL is widely used for producing and processing drug carriers, nanofiber spinning agents, and implantable plastic materials, as it is characterized by excellent shape memory and temperature control properties. PCL, used to develop hemostatic materials, can form compounds with chitosan 100, 133, 134, gelatin 100, 135, collagen fibers 136, and starch 100 to improve the biocompatibility and degradability of the materials without changing or improving the hemostatic properties. Cheng* et al.* used the process of phage incorporation to develop PCL/collagen fibers following the electrostatic spinning technique. They studied rabbit back injury and deep muscle injury models, and the results indicated that the compounds exhibited excellent antimicrobial effects and could be used to achieve rapid hemostasis 136. The PCL material used for the preparation of nanofibers could efficiently cover the maximum contact surface area of wounds. Hu *et al.* fabricated alginate/PCL composite fibers that could be used to dress a mouse back wound model. The material could effectively promote the processes of wound healing and tissue repair. Alginate could provide a moist environment for the wound and promote PCL adhesion 137. The hydrophobic nature of PCL does not promote adhesion. Hence, surface modification with hydrophilic or mucoadhesive materials is required to prepare hemostatic materials. The degradation rate of PCL is slow, and its degradation rate and drug release profile must be considered when it is used as a carrier of hemostatic drugs.

Acrylate polymers exhibit excellent biocompatibility, broad adhesive properties, water resistance, and durability, which dictate the development and application prospects of the materials in the field of medical adhesives 138. Moreover, these materials are easy to formulate. Medical bioadhesives can adhere tightly to tissue surfaces and close wounds. There are two major acrylate-based tissue adhesives: polyacrylate adhesives and α-cyanoacrylate adhesives. Polyacrylate adhesives are extensively used as tissue adhesives and implant-filling materials. These are widely used as filling materials for orthodontics. These also find their applications in the fields of dental repair, artificial joint replacement, and bone defect filling 139. Polymethylmethacrylate (PMMA)-based bone cement is used to treat unstable fractures and in the field of artificial joint replacements. This material significantly improves the firmness of a prosthesis and reduces the degree of long-term loosening of the prosthesis 140. However, PMMA-based bone cement does not exert osteogenic effects to promote bone healing and does not match the mechanical properties of natural bone. These properties hinder their applications in clinical settings. Li* et al.* prepared PMMA-based bone cement-containing active nano-MgO particles (nano-MgO/PMMA), which significantly improved the biocompatibility of the material and significantly increased the expression of osteogenic genes. The use of the material also promoted the production of calcium nodules in bone tissues when applied to a model of critical cranial bone defect model 141. Since 1950, α-cyanoacrylate-based adhesives have been introduced into the medical field, and these are the first and most widely available tissue adhesives. These adhesives can be used to realize rapid and intense bonding. They exhibit excellent biocompatibility and relatively low toxicity. The degree of tissue rejection is also low 142. The α-cyanoacrylate unit contains negatively charged nitrile and ester groups. This makes the double bond in the monomer extremely reactive, and the process of anionic polymerization can be rapidly realized under these conditions under the influence of weak bases. Instantaneous bonding is realized under these conditions while encountering bleeding wounds. Several tissue adhesives, primarily α-cyanoacrylates, are widely used in a variety of surgical procedures. Alfredo Moreno-Egea conducted a randomized controlled trial to explore the effectiveness of n-hexyl-α-cyanoacrylate as a suture substitute in inguinal hernia surgery. The study included 208 patients, 102 of whom were treated with tissue adhesives. The results demonstrated that the use of n-hexyl-α-cyanoacrylate significantly reduced the mean operative time and decreased postoperative pain 143. Cyanoacrylate was effectively used during the endoscopic ultrasound-guided treatment of fundic varices formed under conditions of portal hypertension 144. The α-cyanoacrylate-based adhesive, applied to a dog model with severe liver injury, can close wounds and reduce bleeding 145. Some problems still need to be addressed to increase the application prospects of α-cyanoacrylate-based adhesives. These adhesives may cause foreign body reactions in living tissues and hinder the process of tissue healing. The poor elasticity of the adhesion site and the poor flexibility with the surface tension of the organ limit the practical applications of the adhesive.

PEO is a water-soluble polymer that exhibits excellent biocompatibility, non-immunogenicity, and non-toxicity. It resists the non-specific adsorption of proteins and is frequently used to develop various drug carriers and anti-adsorption coatings 146. In the field of hemostatic material development, PEO is primarily used as an additive to improve the performance of other active ingredients. Chitosan, as a positively charged hemostatic active substance, can promote wound hemostasis by aggregating red blood cells, activating platelets, and activating coagulation cascade reactions 53. The chitosan-based nanofibers fabricated following the electrostatic spinning method adhere tightly to tissues and exhibit excellent biocompatibility and degradability. The application of PEO to the chitosan electrospinning process can effectively reduce the surface tension and viscosity of the chitosan solution, promoting the formation of nanofibers 147. Deineka *et al.* used nanofiber mats formed using PEO and chitosan to study a rat liver injury model, and the composite nanofibers significantly reduced the bleeding time. The material exhibited improved degradability and tissue repair compared to the conventional chitosan sponge 37. The composite nanofibers formed from PEO and chitosan exhibited reduced adhesion on the EpiSkin model. This helped to safely remove the hemostatic material from the wound surface without causing secondary bleeding 148. Poly (propylene oxide) (PPO) is insoluble in water, biocompatible, and stable. It is usually combined with PEO to prepare high-performance materials. Pluronics**^®^** is a PEO-PPO-PEO copolymer that the FDA has approved for usage as an injectable pharmaceutical excipient 149. PEO and PPO do not promote tissue hemostasis or blood coagulation by themselves, and these are mostly explored together with other hemostatic active ingredients for the development of hemostatic materials.

Most synthetic polymers lack the biological activity required to promote blood clotting. These function as substrates or cross-linking agents for hemostatic materials. It is difficult for simple polymers to meet the special performance requirements of biomedical materials. Researchers are widely compounding synthetic polymers with different biologically active natural polymers to integrate their strengths and obtain composite materials that can be effectively used in clinical settings. PU sponge is a porous foam obtained by foaming PU. The interior of the material appears porous, and the material exhibits excellent physical and mechanical properties. Aqueous PU and sulfated chitosan are blended using glutaraldehyde as the cross-linking agent to form a cross-linked semi-interpenetrating network characterized by excellent hemocompatibility 150.

#### Inorganic materials

Inorganic materials can be used effectively for hemostasis. Zeolite has long been noted for its hemostatic properties, and in 2002, QuikClot^®^, the first generation of zeolite hemostatic products, was introduced as first aid equipment for the U.S. military 151, 152. Zeolite is a porous crystalline aluminosilicate, and its porous structure allows for positive water absorption. This helps increase the concentration of local platelets and clotting factors. The negative surface charge can activate positively charged Factor XII, triggering a cascade reaction of endogenous clotting 153, 154. Zeolites not only tend to enter capillaries (resulting in thrombosis) but also tend to absorb water and release excessive heat. This can potentially damage the skin 155. Fan *et al.* prepared calcium-based zeolites that effectively shorten clotting time and help avoid adverse effects. The FDA has approved the patented zeolite hemostatic gauze for use in clinical settings 156.

Mesoporous silica is characterized by a high density of silanol anions on its surface. These anions can interact with the positively charged amino acids on the surface of the coagulation factor XII to accelerate the coagulation cascade reaction and promote hemostasis. Mesoporous silica exhibits good biocompatibility and exerts low degrees of side effects when used in the human body 21. Characterized by the presence of large pores, it can attach to thrombin 157, antimicrobial agents 158, 159, and analgesic drugs 160 to achieve effective wound treatment. Wang *et al.* used silica nanoparticles for loading tannic acid. These materials could effectively promote the activation of the coagulation cascade reaction and reduce the hemostasis time by 65%. These also exhibited excellent antibacterial properties 38. Silica- and chitosan-based composite sponges were used to study a rat tail break model, and these materials exhibited excellent hemostatic properties 14. The positive charge on the chitosan surface and the negative charge of the diatom attracts each other, reducing the amount of powder residue on the trauma surface.

Graphene is readily functionalized and widely used in various biomedical fields, such as drug delivery 161, 162, bioimaging 163, 164, and tumor therapy 162, 165. Graphene oxide (GO) has a 2D sheet structure and contains numerous oxygen-containing functional groups on its surface. These groups can activate platelets to trigger aggregation, and the material exhibits excellent biocompatibility and hydrophilicity. Thus, GO can potentially be used for trauma treatment 40, 166. Graphene-based sponges are characterized by good water absorption abilities, and these are functionalized on the surface. Thus, multiple functional composites can be prepared from GO, laying the foundation for their development as hemostatic materials. Li* et al.* prepared graphene/montmorillonite composite sponges (GMCS) using graphene-immobilized montmorillonite (MMT) particles. GMCS-activated Factor XII with a negative charge cascade on the surface of MMT could be used to accelerate hemostasis, and the entry of MMT particles into blood vessels could be prevented 41.

#### Metal-containing materials

Metal ions such as Ag, Zn, and Cu exhibit broad-spectrum antimicrobial properties, superior heat resistance, good dispersibility, and low drug resistance. These materials are widely used to develop various biological materials and medical devices. Various materials containing metal ions have been effectively used in the field of hemostasis. Ca^2+^ significantly affects the physiological coagulation pathway and can activate Factor X. It participates in the formation of prothrombin complexes and activates Factor XIII to form insoluble fibrin multimers that exert hemostatic effects 167-169. The precipitation of calcium ions on the surface of chitosan nanofibers can effectively accelerate the platelet activation process and shorten the hemostasis time. The composite material prepared by combining hydroxyapatite with bacterial cellulose promotes cell proliferation 170. The calcium-based zeolite can be used effectively to realize hemostasis 156.

Silver ions are antimicrobial agents, and their incorporation into mesoporous silica-chitosan composites can improve the antimicrobial properties of the composites without increasing cytotoxicity 171. Microporous chitosan-polyethylene glycol hydrogels containing copper ions exhibit good stability. These compounds exhibit excellent antimicrobial activity and generate an excellent keratin-forming cell response 172. Zinc is considered a platelet modulator that can promote the process of platelet activation 173. Cellulose oxide nanoparticles loaded with Zn ions can significantly improve the mechanical and antimicrobial properties of bionic calcium alginate hydrogels 174. Carboxymethyl chitosan zinc (CMCS-Zn) is a degradable biomaterial that can be used to treat bone defects effectively, and this composite inhibits the occurrence of bone infections 175. Cerium oxide exerts a significant neuroprotective effect under conditions of oxidative stress 176. *Liu J et al.* synthesized cerium-containing spherical mesoporous bioglass particles, effectively reducing the clotting time and promoting cell proliferation. The results were arrived at by conducting *in vitro* tests 177. Magnesium ions significantly affect the processes of cell adhesion, proliferation, and mineralization 178. Bian *et al.* prepared bioactive hydrogels based on HA and bisphosphonate-magnesium nanoparticles, which could significantly upregulate the production of alkaline phosphatase (ALP) and effectively promote the repair of bone tissues 179. Lu* et al.* loaded Fe^3+^ onto the surface of sodium alginate grafted dopamine-modified poly (lactic acid) (PLLA) complexes. The resulting material could effectively concentrate local blood components and shorten coagulation time. The material exhibited excellent antibacterial properties against *E. coli* and *Staphylococcus aureus* 180. Zinc ions also exhibit significantly high antibacterial activity, and these are used to develop hemostatic materials 18.

### Others

Platelet-derived nano-vesicles and synthetic platelet mimics can be used to develop hemostatic materials. Platelets are small pieces of cytoplasm that are shed from the cytoplasm of mature megakaryocytes. These participate in hemostasis and coagulation. The von-Willebrand Factor (vWF) receptors on the surface of collagen fibers are exposed when vascular endothelial cells are damaged. Glycoprotein Ib (GPIb) on the platelet surface recognizes the bound vWF receptors and rapidly adheres to the site of injury. Activated platelets release epinephrine, ADP, and thrombin, which promote the process of platelet aggregation, resulting in the formation of thrombi. Platelets present a phospholipid surface that adsorbs large amounts of coagulation factors, increasing the speed of the clotting response. Platelet-derived particles can promote the processes of megakaryocyte differentiation and platelet production. Jung* et al.* developed platelet-derived nanovesicles following a hypotonic ultrasound method. The nanovesicles can adhere and aggregate to stop bleeding in the presence of calcium ions and thrombin. Reduced pro-inflammatory cytokines, such as TNF-α, IL-6, and IL-1β, which can potentially hinder the processes of macrophage activation and phagocytosis, are produced under these conditions 43. Platelet mimics can mimic and enhance the hemostatic capacity of platelets. The shelf life is increased, and the degree of contamination is low. These materials can be used to develop hemostatic materials in the future. *Christa et al.* used liposomes as model particles with collagen, vWF binding peptides, and fibrinogen mimetic peptide (FMP) decorated on the liposome surface. The functionally integrated platelet mimetic liposome structure exhibited significantly high hemostatic efficacy when used to study the mouse tail transection model 44. James *et al.* developed Arg-Gly-Asp (RGD)-based functionalized nanoparticle platelet mimics. Poly (lactic-co-glycolic acid)-poly-L-lysine (PLGA-PLL) was used as the core, and RGD sequences were combined with nanospheres via PEG to form the synthetic systems. The material could be used to effectively reduce the bleeding time in a severely traumatized rat model 181. Several biomaterials are also used to develop hemostatic materials. Extracts of lactic acid bacteria combined with nitric oxide donors can be used to form biomimetic phage microparticles exhibiting antimicrobial properties. These microparticles are further encapsulated using graphene oxide, and the resulting antimicrobial hydrogels are prepared to promote the process of wound healing 182. Herbal components such as curcumin have also been investigated for the development of hemostatic materials, and the materials exhibit good antibacterial activity 183.

### Forms of hemostatic materials

The effectiveness of hemostatic materials depends not only on the active chemical composition but also on the selection of the appropriate form of the material to be processed. Nanofibers, hydrogels, nanoparticles, and sponges are common forms of hemostatic materials. Different forms of hemostatic materials are used under different conditions, and the pros and cons of using these materials differ based on the type and form of the material used.

#### Nanofibers

Any fiber with a diameter of less than 1 μm is a nanofiber. These ultrafine fibers have a specific surface area that generates excellent adsorption properties and surface activity. The pore sizes of the lattices and films composed of these extremely fine interwoven nanofibers are significantly small. The materials are characterized by high porosity and good surface adsorption properties. The high porosity of the nanofiber surface helps achieves moderate levels of wound drying, which promotes wound healing. Nanofibers can be classified into two types based on the preparation methods (spinning and molecular technology) followed. In this section, we introduce the application of nanofibers, prepared following the two methods, in the field of hemostatic materials.

**Figure 1:**
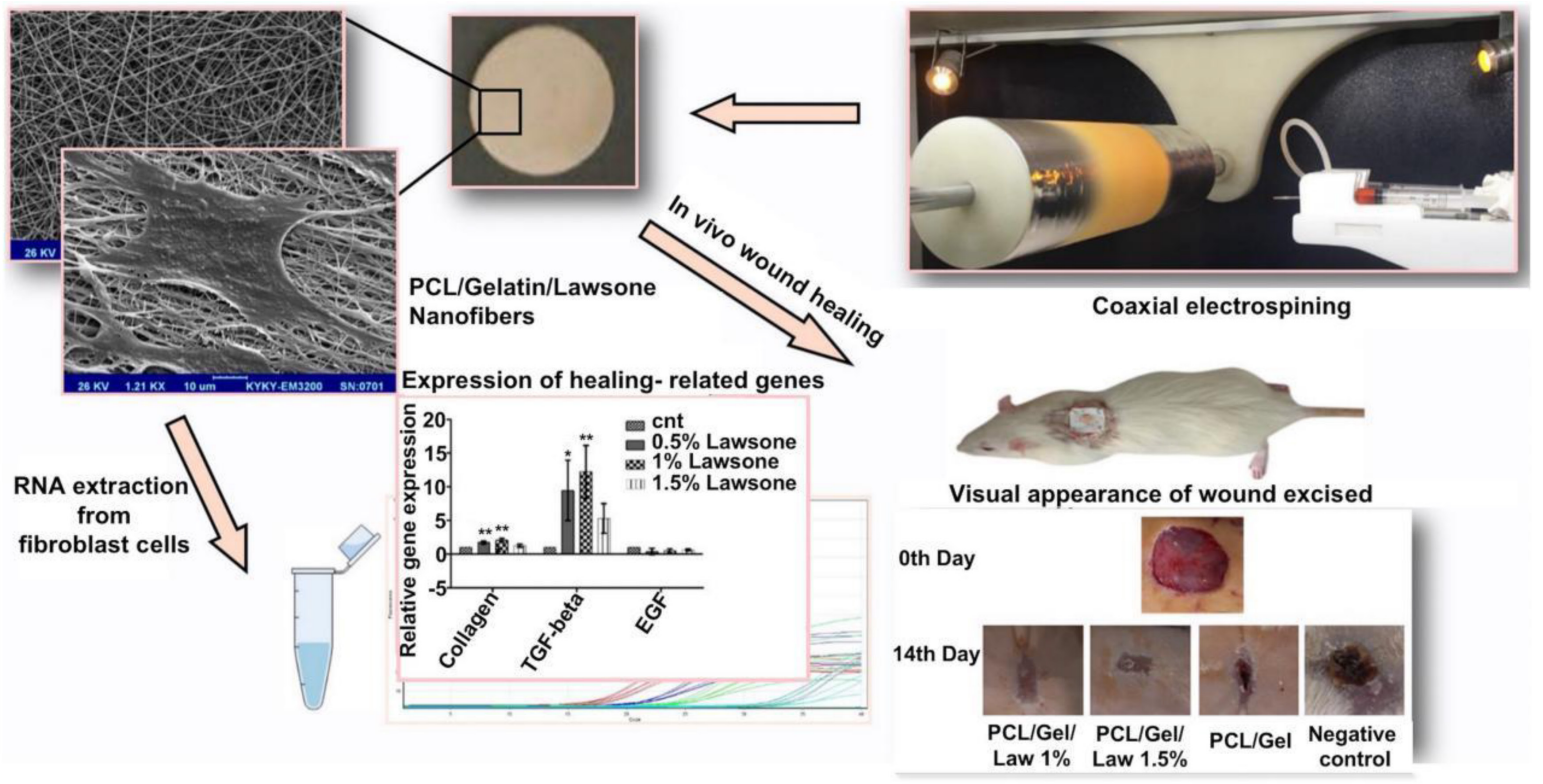
The electrostatic spinning technique was applied to prepare PCL-gelatin nanofibers loaded with different concentrations of Lawsone, and they were experimentally evaluated for wound healing effects in a rat wound model. Reproduced with permission from 34. © 2018 Elsevier B.V. All rights reserved.

Spinning methods include electrostatic spinning 184, solution blowing, and centrifugal spinning 185 methods. The electrostatic spinning method is the most commonly used method for nanofiber preparation as the method is easy to execute. Moreover, the method is widely used in various fields and is characterized by high production efficiency. The electrostatic spinning technology involves the use of the electrostatic force generated under conditions of a high-voltage electric field to prepare the polymer solution extruded from a syringe at a certain flow rate 186. The surface tension is overcome under these conditions, and a fine stream of charged polymer is ejected. The process is influenced by the electrostatic force, and the charged jet further accelerates to stretch and form a continuous ultra-fine fiber that is collected at the receiving end. Sardou* et al.* loaded the nanofibers prepared following the electrostatic spinning method with the PCL/gelatin mixture containing different concentrations of lawsone*.* The results of *in vitro* cell adhesion and proliferation experiments and those obtained by studying rat wound models revealed that PCL/gelatin nanofibers loaded with 1% lawsone can effectively promote the processes of fibroblast adhesion and proliferation. These can also promote wound healing and the formation of granulation tissues 34 (Figure 1). Ahmed* et al.* synthesized nanofiber mats by encapsulating ZnO particles with chitosan/PVA nanofibers prepared following the electrospinning process. Results of *in vitro* experiments revealed the significantly high inhibitory effects exerted by chitosan/PVA/ZnO nanofibers on a variety of bacteria, including *Staphylococcus aureus*, *E. coli*, and *Pseudomonas aeruginosa*. The chitosan/PVA/ZnO nanofibers exhibited strong antioxidant capacity in a diabetic rabbit wound model, inhibiting the development of local inflammation and promoting wound healing 18. The polymer solution should be characterized by a certain level of conductivity for the efficient preparation of nanofibers following the electrostatic spinning method. This limits the choice of conductive polymers. Moreover, the electrostatic spinning method cannot be used for industrial production, as the products are obtained in low yields when this method is used. This resulted in the development of the solution spinning technology. Unlike the electrostatic spinning method, the solution-blowing spinning method involves the exploitation of the jet effect generated by high-pressure airflow when droplets overcome surface tension to form nanofibers 185. The solution blow spinning technique is a highly efficient method that can be used to prepare materials within a short time. Electrostatic interactions and dielectric constants do not limit the process. Daristotle* et al.* prepared a mixture of solutions containing PLGA, PEG, and SiO_2_ nanoparticles with acetone as the solvent and deposited polymer nanofibers on a porcine liver wound model using a solution blow-spinning device. The results demonstrated the good hemostatic effect exerted by the nanofibers 36. It should be noted that the fibers are formed randomly, and the expected morphology is not achieved when the solution spinning technology is used. Xie *et al.* manufactured nanofiber capsules following the processes of electrospinning, gas foaming, coating, and cross-linking. The capsules can be swallowed. Good scalability and absorption properties are achieved, and the materials are promising as alternative testing products to endoscopy that can be used to collect a large number of biological samples to help diagnose numerous diseases in a timely and accurate manner 187.

Molecular techniques are primarily used to prepare single- or multi-tube carbon nanotube (CNT) bundles. The three main methods used for the preparation of CNT bundles are arc discharge 188, laser ablation, and chemical vapor deposition 189. Among these, the chemical vapor deposition method is the most popular as it is cost-efficient. This method can be used to produce materials with high yields, and the process can be executed under controlled experimental conditions. The chemical vapor deposition method is used to deposit catalysts on a flat substrate such as quartz. The method involves the passage of a carbon-containing gas at a high temperature to decompose, precipitate, and grow CNTs on the catalyst particles. This method can be used to produce ordered parallel/perpendicularly aligned CNTs that are characterized by high specific surface areas 189. Patel *et al.* modified the surface of polymeric nanofibers with CNT and investigated the role of the composites in modulating inflammation, angiogenesis, and bone repair (*in vivo*). The CNT-coated nanofibers exhibited a reduced inflammatory response and enhanced angiogenic and angiogenic marker expression levels when injected subcutaneously into rats 35. The electrical conductivity of CNTs can also induce osteoblast differentiation and cell proliferation through electrical stimulation, and these materials are frequently used to prepare synthetic bone matrices 190. The major obstacle that hinders the further application of CNTs in biomedical fields is their toxicity toward tissue cells 191.

The nanofibers of natural or synthetic polymers prepared following the above methods can effectively adhere to and activate platelets to promote the processes of hemostasis and wound healing in cases of serious or irregular injuries. However, hemostatic materials are difficult to remove, and this problem is yet to be addressed.

#### Gels

##### Hydrogels

Hydrogel presents a three-dimensional network consisting of a macromolecular backbone and side chains containing hydrophilic or hydrophobic groups. It can absorb and retain large amounts of water by exploiting the cross-linked network. Its application in the field of hemostasis and wound healing can increase the concentration of local platelets and clotting factors and shorten the clotting time 192. Most existing hemostatic materials adhere weakly to wet tissues and cannot be effectively used to treat heavy-bleeding wounds. Hydrogels can effectively adhere to tissues, keeping the wound site moist to stop bleeding and promote healing. The hydrophilic groups in the hydrogel can bind tightly to the local protein or peptide units in tissues. The hydrogel adheres firmly to damaged tissues under these conditions 193. Hydrogels can be classified according to the preparation method, source of the raw material, size of the material, and shape of the material. Herein, we discuss hydrogel-based hemostatic materials that are categorized based on the preparation method.

Hydrogels can be classified into physical and chemical hydrogels based on the hydrogel synthesis method followed and the nature of network bonding. Physical hydrogels are formed under the influence of physical forces such as electrostatic interactions, hydrogen bonding, and chain entanglement 194. Physically prepared hydrogels are prepared in the absence of chemical cross-linking agents. They are readily degradable and can be used extensively used in the biomedical field. These materials are characterized by good degradability and thixotropy. Physical hydrogels can be converted to solutions when heated; therefore, these are also known as pseudogels or thermally reversible gels 195. The PVA hydrogel obtained following the repeated freezing-thawing process is a physical hydrogel. The distribution of the PVA molecular chain in the homogeneous aqueous solution of PVA is highly disordered. The mutual contact between the chains is limited, and this makes the formation of cross-links difficult 196. The freezing process helps freeze the movement of the PVA molecular chains for a moment. This helps increase the contact time. The van der Waals forces and hydrogen bonds are exploited to form tightly bound local physical cross-linking points. The repeated freezing-thawing process results in the formation of a plurality of physical cross-linking points in the PVA hydrogel. Heating destroys the physical cross-linking point between the molecules, and the PVA molecular chains revert to the disordered state. The hydrogel changes to the liquid state under these conditions. The heating method is one of the most common methods used for the preparation of thermosensitive polymer gels. Xu* et al.* prepared self-assembled graphene hydrogels (SGH) by heating aqueous dispersions of GO in a Teflon-lined autoclave. The samples were heated at 180 °C for 12 h 197. The SGH obtained following this method is characterized by excellent mechanical strength and thixotropy. It can be easily prepared. It is noteworthy that the high-heat reaction is hazardous. Electrostatic interactions can stabilize hydrogel networks. Metal cations (Ca^2+^, Mg^2+^, and Fe^3+^) can interact with negatively charged polysaccharide carboxylate groups to form tight networks. This results in the generation of mechanically sound metal-polysaccharide hydrogels. Polysaccharide polymers capable of forming cross-linked hydrogels with metal ions include chitosan 19, 198, pectin 199, and cellulose 200. The hemostatic effect of the polysaccharide moieties helps maintain moisture in wounds and promotes the process of wound healing. The physical methods used to prepare hydrogels are reversible, and the hydrogels are formed under mild conditions. It has been observed that the stability and mechanical strength of such hydrogels are poorer than those of the hydrogels prepared following other methods.

Hydrogels prepared following chemical methods (for example, those prepared in the presence of cross-linking agents or those synthesized by accelerating the reaction of functional groups in polymer chains) present a stable structure. These are widely used in the field of biomedicine 113. PVA-based hydrogels can be synthesized using cross-linking agents and following the freezing-thawing method described in the previous section. The addition of glutaraldehyde to the aqueous solution of PVA induces the alcohol-formaldehyde condensation reaction that results in the cross-linking of PVA to form a network of polymer hydrogels 201. Such cross-linking agents are integrated into the hydrogel cross-linking network. The use of a type of zero-length cross-linker allows the formation of covalent bonds between polymer chains. Integration of the system into the hydrogel network is avoided under these conditions. For example, 1-ethyl-3-(3-dimethylaminopropyl) carbodiimide (EDC) can react with a carboxylic acid group on the polymer chain to form an o-acyl isourea unit. Urea is formed as the by-product, and it is subsequently released when a primary amine attacks the reactive ester intermediate on the adjacent polymer chain. The reaction results in the formation of a hydrogel network that is stabilized by covalent amide crosslinks 202. Such cross-linking agents are primarily used to prepare gelatin- 203, collagen- 202, and fibrin-based hydrogels. The mixing of nanoparticles and polymers induced the formation of hydrogel cross-linked networks. The interaction between hydrophobically modified carboxymethylcellulose-based polymer chains and polystyrene or PEG-based nanoparticle surfaces facilitates the formation of polymer-nanoparticle hydrogel networks. The reversible hydrophobic forces under action allow the hydrogel to flow under the influence of the applied shear stress. When the stress is relaxed, the hydrogel regains its original properties 204. The hydrogel produced from the nanoparticle blend can be effectively used for drug delivery, and it is currently used for the treatment of subcutaneous melanoma in a mouse model 205. Some enzymes facilitate the hydrogel cross-linking reaction. Transglutaminase catalyzes the formation of isopeptide bonds between lysine and glutamine side chain amides. This reaction can be used to produce intermolecular crosslinks between soluble fibrinogen molecules, resulting in the formation of fibrinogen- and fibrin-based hydrogels 206. Lysyl oxidase, a copper-dependent enzyme associated with the formation of collagen fibrils, allows the formation of aldehydes from lysine side chain residues under conditions of enzymatic reduction that is followed by the process of interpeptide cross-linking (such as Schiff base reaction). The process results in the production of covalent hydrogels 207. UV light triggers the process of gelation through the action of photoinitiator molecules which absorb radiation and form substances that can mediate polymerization reactions 208. The photo-initiator is irradiated by UV light, and this results in the formation of free radicals which attack the vinyl group. Under these conditions vinyl free radicals are formed and the polymerization of the vinyl group proceeds under conditions of free radical polymerization. Therefore, most of the base materials commonly used to prepare photo-cross-linked hydrogels are vinyl-functionalized polymers, such as PEGDA 209, gelatin methacrylate (GelMA) 210, and methacrylated HA (HAMA) 211. The cross-linked network of hydrogels formed in the presence of photo-initiators is uncontrolled, and heterogeneous networks are produced under these conditions.

**Figure 2:**
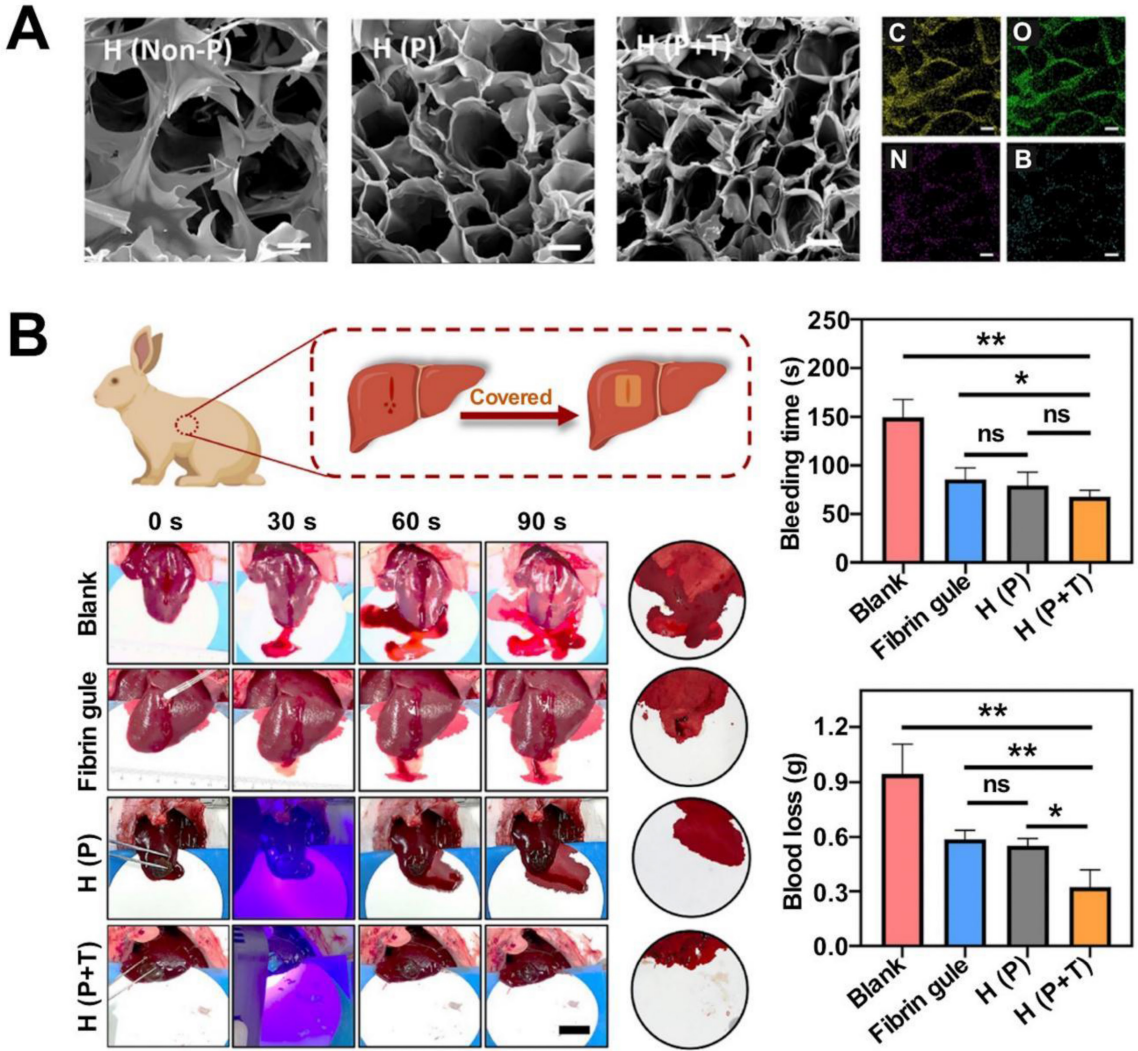
Multifunctional biohydrogels based on carboxymethyl chitosan, sodium alginate and tannic acid and its application in rabbit liver injury model. A: scanning electron microscope images of hydrogels and elemental distribution of C, N, O and B; B: Schematic diagram of the application of hydrogel in a rabbit liver hemorrhage model, including photos of the hemostatic process, comparison of bleeding time and blood loss. Reproduced with permission from 214. © 2022 Zou Chen-Yu* et al*.

Hydrogel, as a hemostatic material, adheres strongly to tissues and can be used to treat wounds with high blood flow. The Schiff base reaction is a nucleophilic addition reaction between primary amine-bearing compounds and carbonyl-containing aldehydes and ketones 212. The adhesive formed from cross-linked hydrogels produced following the Schiff base reaction presents a network-like structure that is characterized by a high degree of porosity and water content. These materials are biocompatible and readily bind to tissues. The adhesive strength of the hydrogel is high, and the material can be potentially used to heal wounds 213. Yu* et al.* attached *O*-nitrobenzyl alcohol (NB) units (modified under photosensitive conditions) to the backbone of water-soluble carboxymethyl chitosan (CMC), exploiting amide bonds to synthesize the NB-CMC composite. Under conditions of UV irradiation, the NB units in the NB-CMC composite were converted to the *O*-nitrosobenzaldehyde group, and the formaldehyde group reacted with the amino group in CMC to form a stable cross-linked network. Photoluminescent aldehydes react with amino groups present on the tissue surface to form covalent bonds at the hydrogel-tissue interface. Rapid tissue integration is achieved under these conditions, the adhesive strength realized is high. Experimental results revealed that the adhesive strength of the composite hydrogels is significantly higher than that of commercially available bioadhesives used in clinical practice. The former exhibits excellent blood cell adhesion and coagulation ability and can be used to exert excellent hemostatic effects *in vitro* and *in vivo* 20. The multifunctional bioadhesive hydrogels (containing carboxymethyl chitosan (CMCS), sodium alginate (SA), and tannic acid (TA)) prepared exploiting dynamic covalent bonds (borate ester bonds and Schiff base bonds) helped optimize the mechanical, antibacterial, and antioxidant properties of the hydrogels. The improved adhesion ability of the material was obtained using a rabbit liver hemorrhage model 214 (Figure 2). Gelatin hydrogels are cross-linked via amide bonds, and the cross-linking process usually proceeds following the EDC-based coupling method. The amide bond is formed between the primary amine and the carboxylic acid group on the side chain of the adjacent gelatin chain. Ya* et al.* introduced cyclized succinate groups into a tetra-PEG hydrogel. The residual butadiimide active ester in the compound could react with the amino group in the tissue to form stable amide bonds. This helped to seal the wounds and control bleeding 99 effectively. Experimental results revealed that the tetra-PEG hydrogel could withstand pressures of ≤294 ± 27 mmHg, and the material exerted significantly high levels of hemostatic effects on a porcine spleen hemorrhage model. The challenges lay in removing the hydrogels that exhibited strong adhesion properties.

Hydrogels are a popular form of hemostatic material. The residual aldehyde group on the surface of hydrogels prepared following the Schiff base reaction is potentially cytotoxic, and the strong adhesion ability of the hydrogel makes the safe removal (without causing pain to the patient) of the hydrogels challenging.

##### Aerogels

Aerogel is a nanoscale porous solid material formed by replacing the liquid phase in the gel with gas following a drying method. Aerogel is the least dense solid in the world and is characterized by high porosity, specific surface area, and adsorption power. Aerogel accelerates hemostasis by absorbing local water from wounds. The porous structure of the aerogel and an increase in the concentration of clotting factors and platelets promote water absorption. A substrate for clot attachment is formed when the aerogel comes in contact with blood. Aerogels can be classified into three categories based on the backbone constituents: inorganic aerogels, organic aerogels, and carbon aerogels.

Inorganic aerogels are currently being researched for hemostasis, and the most common material being studied is silica. The sources of raw materials used to prepare SiO_2_-based aerogel are abundant. The preparation process followed is simple and good controllability is achieved. These aerogels are characterized by high porosity and high specific surface area. The freeze-drying method is the most common method that is followed to prepare aerogels. The microscopic pore structure of aerogels can be modified by controlling the cooling rate and temperature gradient. Yin* et al.* reported that an increase in the cooling rate results in an increase in the degree of refinement of ice crystals. This eventually results in the formation of a fine and homogeneous pore structure 215. Chen* et al.* prepared chitosan/diatom biomineralized silica-based aerogel systems following the freeze-drying method. The pore size of the material could be controlled. The composite aerogel presents a hierarchical porous structure. The ice crystals formed using *tert*-butyl alcohol during the freeze-drying process can maintain the porosity of biomineralized silica in the aerogel. Under these conditions, a composite aerogel with a high specific surface area (74.441 m^2^/g) and excellent water absorption ability (316.83 ± 2.04%) is formed. The hemostatic time recorded for the composite aerogel was less than 70 s (*in vitro*), and the material outperformed similar hemostatic products in terms of hemostatic time and bleeding volume indices. The results were arrived at by studying the rat severed tail model and the femoral arterial vein dissection model 21. Metal oxides also constitute an inorganic skeleton of aerogel. Oxide-based aerogels such as TiO_2_, ZrO_2_, and Al_2_O_3_ are used primarily to prevent air and water pollution. Electrically conductive oxide-based aerogels have also been developed 216, 217.

**Figure 3:**
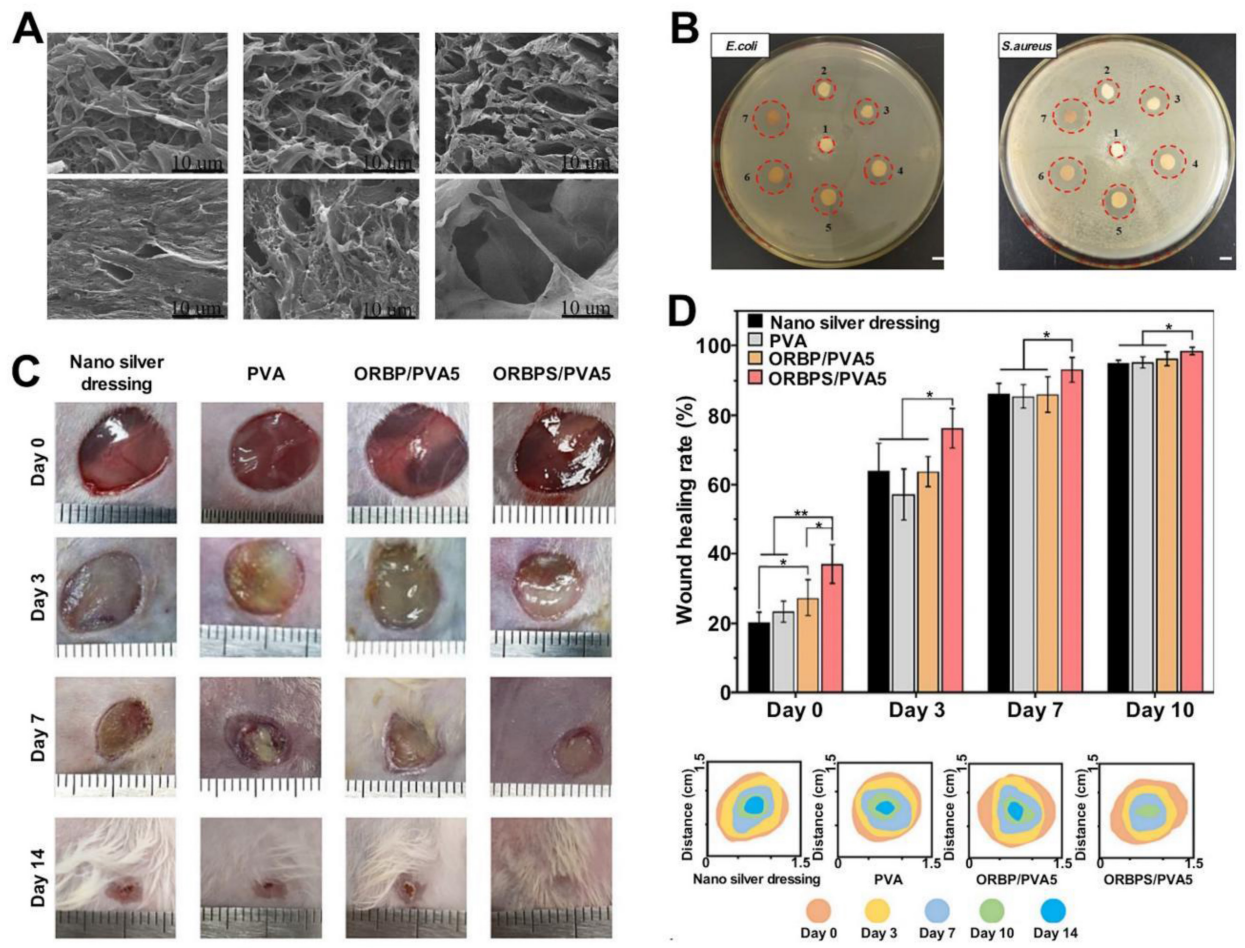
Antimicrobial and wound healing properties of oxidized Bletilla rhizome polysaccharide-based aerogel. A: scanning electron microscopy images of porous aerogels; B: antibacterial activity of aerogels against *E. coli* and *S. aureus*; C: application to a mouse model of total skin defect to promote wound healing and D wound healing rate statistics (comparison with nano silver dressing). Reproduced with permission from 32. ©2022 Elsevier Ltd. All rights reserved.

Organic aerogels can be classified into two categories: biomass-based and polymer-based. Biomass-based aerogels are biocompatible, biodegradable, and widely used in the pharmaceutical and food industries. Various plant and animal biopolymers have been used to prepare aerogels. Saini *et al.* prepared a biomass-based organic aerogel by freeze-drying a viscous gel obtained by mixing microprotofibrillated cellulose (MFC; prepared from cinnamon extracts) and carboxylated cellulose nanocrystals (cCNCs). The compressive strength of this aerogel was approximately 106% higher than the compressive strength recorded for MFC. The modulus of the former was 175% higher than that of the latter 218. Aerogels based on carbon fibers (CNFs) can be prepared following the processes of freeze-drying, supercritical drying (SCD), spray drying (SD), and oven drying (OD) 219, 220. Freeze-drying at -80 °C and 15 Pa for approximately 72 hours helps obtain a mixture of poly(amidoamide) epichlorohydrin resin and CNF. The SD method is used to concentrate and extract liquid from the CNF-based hydrogel. The sample was dehydrated in hot gas. The primary advantage of using this preparation method lies in the fact that the size of the aerogel pores can be controlled effectively using the SD method, and the preparation process is cost-effective. CNF-based aerogels exhibit excellent water absorption and adhesion properties. Wang* et al.* prepared a composite aerogel consisting of CNF and copper-containing mesoporous bioactive glass. The prepared aerogel exhibited excellent antimicrobial properties and could inhibit the growth of *E. coli* and other inflammatory bacteria. This could be potentially used to treat chronic wounds 42. Polymer-based organic aerogels are polymer molecules with porous network structures. These aerogels are prepared by combining colloidal particles by exploiting hydrogen bonding or van der Waals forces. Yan* et al.* prepared an antibacterial aerogel-based dressing using PVA and oxidized betaine polysaccharide Schiff base (ORBPS). The material was fabricated following the freeze-drying method, and a mouse model of total skin defect was studied. The dressing was found to reduce inflammation, promote angiogenesis, and facilitate the wound-healing process 32 (Figure 3).

Carbon aerogels are chemically stable materials characterized by high hardness and melting points. The carbon skeleton structure is solely retained under an inert atmosphere and high-temperature conditions. These aerogel systems are primarily used in fields operating under high-temperature conditions. Carbon aerogels find their use in the field of aerospace engineering and high-temperature furnaces. These are also used to produce nuclear energy. Graphene aerogel is a special type of carbon aerogel. GO is usually reduced and treated to obtain graphene gel, which is freeze-dried or supercritically dried using CO_2_ to obtain graphene aerogel. GO presents a 2D lamellar structure and contains numerous oxygen-containing functional groups on its surface. It can activate platelets and trigger a strong aggregation reaction, promoting hemostasis. Jessica* et al.* prepared aerogels based on GO and gelatin and experimentally demonstrated that the sample could absorb 50 times its weight when in contact with liquid media under physiological conditions. Analysis of the SEM images revealed that red blood cells adhered well to the sample surface 40. The use of highland anthocyanin (concentration: 5%) can effectively improve the blood absorption and adhesion capacity of the composite aerogel, and the negative charge on the surface of highland anthocyanin can promote the process of platelet and erythrocyte aggregation exploiting electrostatic interactions.

The highly porous structure of aerogels has attracted the attention of researchers working in the field of drug delivery 221. The unique structure enables the rapid loading of small-molecule drugs and provides a low degree of restriction to the internal regions of the matrix. Site specificity, stimulus responsiveness, and prolonged drug release properties are also achieved.

#### Sponges

Sponges exploit their dense porous structure to absorb local moisture from wounds. This results in an increase in the concentration of platelets and clotting factors. The platelets adhere and aggregate under this condition, forming a thrombus that stops bleeding. Absorbent gelatin sponges are the most frequently used hemostatic material. Commercial gelatin sponges (such as Gelatin Sponge®) are cheaper than other materials and exert a hemostatic effect. These materials can be used effectively to treat deep wounds and trauma. These are also used during surgery, but the poor mechanical properties of the material limit their application prospects. The material is prone to breakage when it encounters blood. It is difficult to stop large wounds from bleeding as it is easy to stretch fragile blood cells and platelets. The freeze-drying method is used to lyophilize the solution and form porous sponges. Alkylated chitosan promotes blood clotting when inserted into blood cell membranes 59. A composite sponge characterized by a porous structure can be formed by lyophilizing a mixture of alkylated chitosan and silica 14. The introduction of different amounts of alkyl groups on the chitosan surface and the different concentrations of alkylated chitosan affect the porosity of the sponge samples. The solution of 1% alkylated chitosan dissolved in acetic acid mixed with silica was lyophilized to obtain a sponge with a porosity of approximately 79%. A high compressive strength was recorded for the sponge sample, and it was observed that the sponge exhibited excellent hemostatic ability when used to study a mouse tail break model.

The pore structure of the sponge also offers the possibility of loading other hemostatic active ingredients. Goncharuk *et al.* prepared poly (vinyl formal) (PVF) solutions by condensing PVA with formaldehyde. Multivacancy sponges were obtained by freeze-drying the mixed solutions. It was observed that the pore size of the samples was affected by the concentration of PVA in the solution 33. PVF sponges consist of large, interconnected pores, the walls of which consist of fine pores of small radii (1-3 μm). The radius of the coarse pore is approximately 430 μm, and that of the fine pores is in the range of 195-225 μm. Chitosan hydrogel, CaCO_3_ particles, and silica particles were added into the coarse and fine pores of the PVF sponge to compare their mechanical strengths, swelling performances, and hemostatic effects. It was demonstrated that the chitosan hydrogel-modified PVF sponge containing fine pores exhibited excellent water absorption ability, and the hemostatic efficiency of the PVF sponge containing fine pores was 92.7% higher than that recorded for Celoxin^®^. The mouse tail break model was used to arrive at the results. However, the application of functional fillers did not significantly improve the hemostatic efficacy of the PVF sponges. This can be potentially attributed to the deactivation of the aldehyde group on the PVF surface. The deactivation could be attributed to the interaction of the aldehyde group with the amino group of the active ingredient. Besides, the potential cytotoxic effect of the residual aldehyde groups should be considered in practical applications.

**Figure 4:**
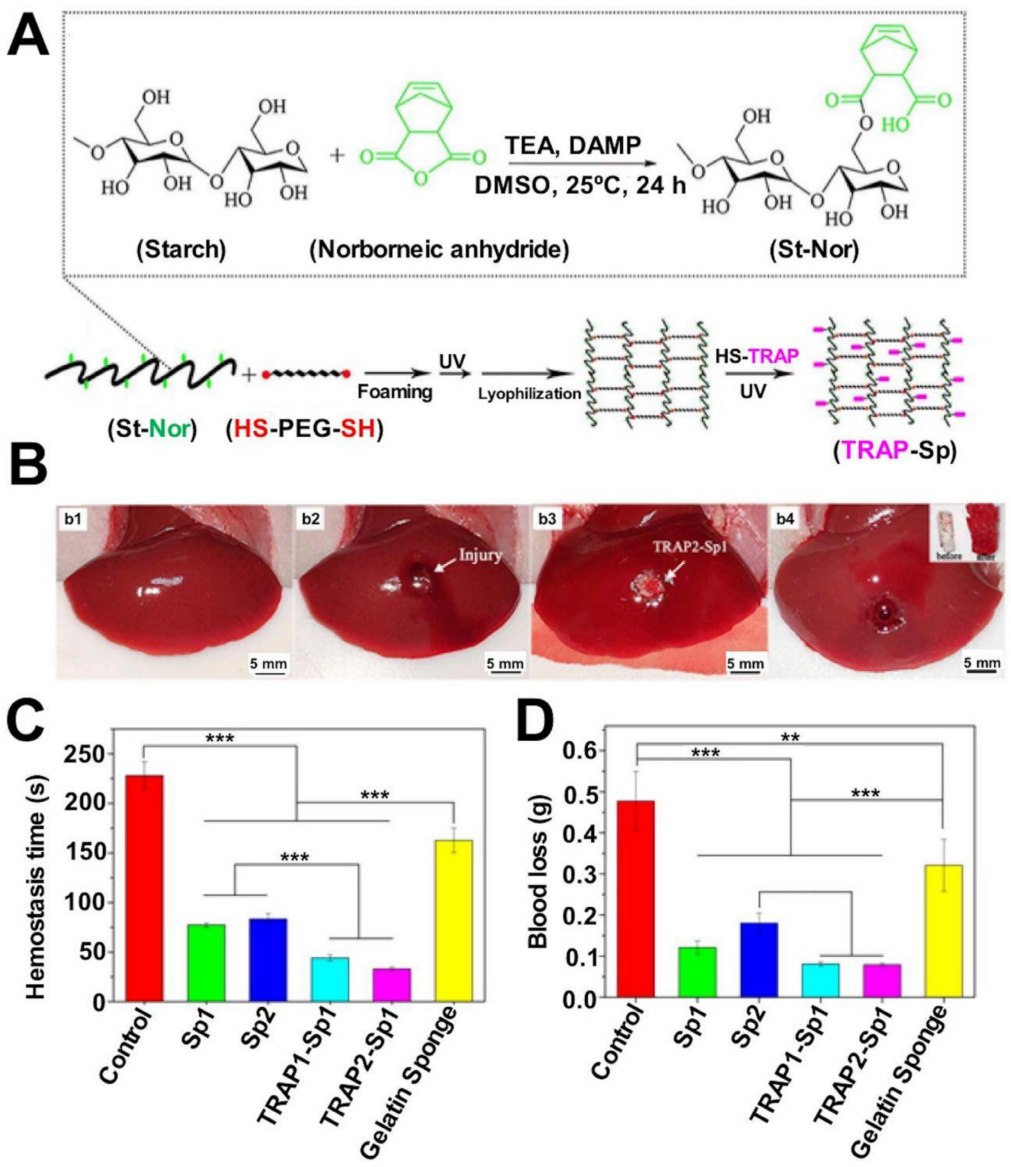
Peptide immobilized starch/PEG sponge for hemostasis in a rat liver defect model. A: schematic diagram of the synthesis of the receptor agonist peptide (TRAP)/PEG sponge; B: schematic diagram of the procedure for hemostasis experiments in liver defects; C: hemostasis time and D: blood loss of the composite sponge in all samples of the rat liver defect model. Reproduced with permission from 29. ©2019 Acta Materialia Inc. Published by Elsevier Ltd. PEG: polyethylene glycol; TRAP: the synthesis of the receptor agonist peptide.

Hemostatic active ingredients such as thrombin receptor agonist peptides (TRAP) can be immobilized on the surface of the sponge to accelerate hemostasis. A modified starch solution and the dithiol-functionalized PEG solution are mixed in a certain ratio, a cross-linked network is established using UV light, and the composite solution is lyophilized to obtain a starch-based sponge 29 (Figure 4). TRAP was immobilized on the sponge surface through thiol-ene click chemistry exploiting sulfhydryl groups. Experimental results revealed that the composite sponge was characterized by a high degree of porosity, and the property of rapid water-triggered shape recovery was also observed. The hemostatic ability of the gelatin sponges and the TRAP-composite sponges were compared using the rat femoral artery and liver defect models. The results revealed that the TRAP-composite sponges could significantly reduce the bleeding volume and accelerate the clotting time in incompressible and arterial bleeding cases. Deep irregular wounds (such as those caused by liver injury) were studied, and it was observed that the TRAP-composite sponge could rapidly expand and close the wounds after absorbing water. The samples were characterized by high mechanical strength and were not significantly toxic to tissue cells.

Sponges could increase the local concentration of platelets and clotting factors in the wounds, and it could be attributed to the high porosity of the sponges and the excellent water absorption properties of the samples. A hemostatic sponge can compress the local area when it swells with water. However, hemostatic sponges are not suitable for the treatment of deep and irregular wounds, and these may cause pain and exert localized pressure on nerves.

#### Nanoparticles

Nanoparticles can be potentially used to treat deep and irregular wounds. These can also be used to load other hemostatic active ingredients or drugs. The use of nanoparticles can ensure the stability and solubility of the loaded ingredients or drugs, promote transmembrane transport, and significantly improve hemostatic efficacy. Nanoparticles formed from natural and synthetic polymers exhibit good drug-loading stability and are easily surface-modified. Controlled release and targeting of drugs can be achieved by modulating the properties of polymers and by realizing surface modifications. Nanoparticles are classified into several categories according to their chemical composition: liposome-based nanoparticles (LNPs), polymeric nanoparticles, and inorganic nanoparticles.

LNPs are extensively studied as drug delivery carriers. These nanoparticles generally comprise ionized cationic lipids, phospholipids, cholesterol, and polyethylene glycol lipids. LNPs exploit the properties of phospholipid molecules to penetrate cell membranes, laying the foundation for the development of drug delivery carriers. Chen* et al.* used LNPs to wrap hemostatic macromolecules such as thrombin and studied the activation of platelets in the presence of LNPs loaded with thrombin. They conducted *in vitro* platelet contraction assays and thromboelastography tests to arrive at the results 222. It was observed that platelet endocytosis of LNPs loaded with thrombin significantly increased the sensitivity of platelets toward collagen and significantly accelerated the time to clot formation. An increase in clot stiffness was also observed. Its introduction into the plasma of patients suffering from coagulation disorders also markedly accelerated the time of formation of blood clots. Attention should be paid to the fact that thrombin not fully encapsulated by LNPs may activate platelets prematurely, resulting in rapid platelet activation and thrombosis. LNPs equipped with ADP could also effectively activate and aggregate platelets, thus significantly shortening the clotting time in a rabbit liver defect model. Thrombus formation in arteries is avoided under these conditions 222.

**Figure 5:**
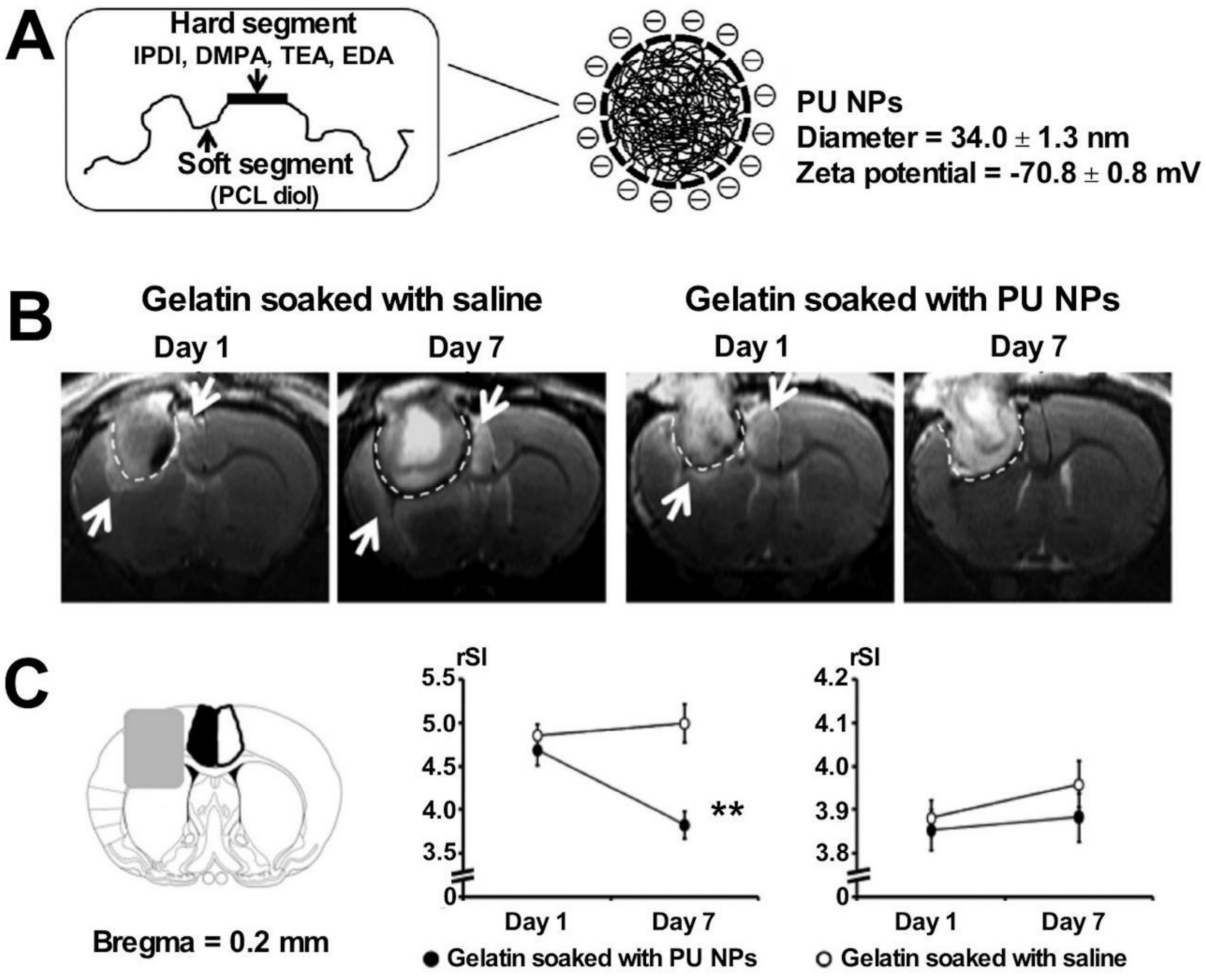
PU NPs allow for hemostasis of fragile brain tissue. A: structure and properties of polyurethane NPs; B: comparison of brain edema formation after implantation of gelatin soaked with saline or PU NPs in a rat neurosurgical model; C: measurement of T2WI signals in the prefrontal lobe and comparison of signal intensity between the ipsilateral and contralateral prefrontal cortex on day 1 and day 7 after implantation. Reproduced with permission from 229. ©2020 Elsevier Ltd. All rights reserved. PU: polyurethane.

Polymer-based nanoparticles can be primarily classified into two categories: natural polymer-based nanoparticles and synthetic polymer-based nanoparticles. The chitosan surface contains a positive charge and can be self-assembled with negatively charged polyanions (such as sodium tripolyphosphate) to synthesize chitosan nanoparticles exploiting electrostatic interactions 223, 224. The size, diameter, and hydrodynamic characteristics of chitosan nanoparticles are influenced by the chitosan concentration, the ratio of chitosan to anionic crosslinker, and the pH of the reflective system. Small chitosan nanoparticles are characterized by better antibacterial properties than large chitosan nanoparticles. The former exhibit better membrane penetration ability than the latter 225. Nanoparticles are smaller than chitosan nanofibers, chitosan hydrogels, and chitosan sponges, and the degrees of dispersion realized for the nanoparticles are higher than the degrees of dispersion realized for the other systems. It has been observed that nanoparticles can effectively treat deep and irregular wounds. These can also function as drug delivery vehicles to realize the precise release of antitumor drugs 226 and insulin 227 to target areas. Some researchers have reported the cytotoxicity of chitosan nanoparticles. Nanoparticles can also be used to treat fragile brain tissues for hemostasis as they exhibit good membrane penetration ability. Moreover, they can also cross the blood-brain barrier. These also exhibit anti-inflammation properties. PU nanoparticles, which differ from most nanoparticles in terms of stimulating immune responses, reduce the gene expression levels of pro-inflammatory factors in macrophages and exhibit the suppressive properties of immune responses 228. Abel* et al.* blended a commercial gelatin granule formulation (Spongostan^®^) with PU nanoparticles and applied it to a rat neurosurgical model. It was observed that the sample significantly accelerated the clotting time and reduced bleeding 229 (Figure 5). Results obtained using the MRI technique revealed that the application of mixed nanoparticles significantly reduced brain edema and neuroinflammation in rats. It is necessary to pay attention to the possible cytotoxicity of the organic solvents used during the preparation process of polymeric nanoparticles.

Most inorganic nanoparticles are easily synthesized. These exhibit good biocompatibility and are compatible with various ligands and biomolecules. Silver ions have antibacterial properties. Małgorzata* et al.* deposited Ag nanoparticles on the surface of chitosan nanofibers prepared following the electrostatic spinning technique. They studied the properties of these fibrous scaffolds without Ag nanoparticles and tested the *E. coli* and *S. aureus* inhibiting abilities 171. Silicon dioxide-based nanoparticles (SNPs) have been the focus of research in the field of drug delivery as they exhibit excellent biocompatibility and are readily functionalized. SNPs could increase the concentration of platelets and coagulation factors by absorbing local water from wounds, and the negative charge on their surface could activate Factor XII to accelerate the coagulation cascade 157. Chen *et al.* prepared functionalized SNPs following the sol-gel method using chitosan and hydrocaffeic acid. They demonstrated that the composite SNPs could effectively accelerate the formation of blood clots and help in hemostasis. They studied the rat femoral artery hemorrhage and the liver injury models to arrive at the results. Results from *in vitro* experiments indicated that the chitosan-modified SNPs could effectively adhere to erythrocytes and connect with fibrin to form blood clots 39.

Chen* et al.* exploited *E. coli* cytoplasmic membranes (EMs) and tumor cell membranes (TMs) from autologous tumor tissues to wrap polymeric PLGA nanoparticles on the surface to study the applicability of the samples in the field of development of tumor vaccines. It was observed that the samples could specifically elicit good anti-tumor reactivity, and significantly high levels of adverse effects were not observed 230. Tests were conducted in mice models inoculated with CT26 colon cancer cells, B16-F10 melanoma, and EMT6 mammary tumors, and the results revealed that the composite nanoparticles could effectively inhibit the recurrence of tumor cells. These were also found to be hypersensitive to immunotherapy.

Hemostatic materials in the form of nanoparticles can be used to stop bleeding from deep and irregular wounds. These materials are portable and exhibit temperature-independent properties. It is noteworthy that nanoparticles are not easily removable. However, substances that are difficult to metabolize and degrade are potentially toxic and may obstruct blood vessels.

### Others

In addition to the single forms of hemostatic materials discussed in the preceding sections, composite hemostatic materials have been developed to take advantage of the strengths of the single forms of hemostatic materials. The disadvantages of the single forms can be negated using composite hemostatic materials. The sponge deposited using gelatin nanofibers is an example of an efficient hemostatic material. Gelatin is spun electrostatically to produce nanofibers, and the continuous built up of the nanofibers results in the formation of a fluffy nanofiber sponge 25. This highly fluffy nano-fiber sponge is ultra-lightweight and is characterized by excellent compressibility. The degree of compressibility is higher than the degree of compressibility recorded for the normal gelatin sponges. The preparation method is easy to execute, relatively cost-effective, and suitable for industrial production. The gelatin nanofiber-based sponge can remarkably reduce bleeding volume and shorten the clotting time in rabbit ear artery bleeding and liver injury models. It can rapidly fill the deep part of the wound attributable to incompressible injury (e.g., liver injury) to plug the bleeding site.

Cryogel presents an interconnected macroporous structure, and it is a shape memory hemostatic material. However, the mechanical strength of cryogels formed at low temperatures is lower than the mechanical strength of the cryogels formed at high temperatures. The introduction of CNT as an additive to cryogels can effectively improve their mechanical properties and the antibacterial ability of the systems 22. CNT was dispersed into the quaternized chitosan solution under the action of ultrasonic waves, and the cryogel with shape memory was obtained under conditions of low-temperature polymerization at -20 ℃ 22. The excellent hemostatic effect was observed in the mouse liver injury and rabbit liver injury models, and the sample could effectively promote wound healing in the whole skin defect model.

Injuries, especially battle wounds or trauma, hemorrhage, and these frequently get infected. Ci *et al.* synthesized biocompatible antimicrobial hybrid nanofibers using MoS_2_/ZnS and PCL following the electrostatic spinning method. The fabricated material exhibited promising antimicrobial activity under photocatalysis and could be potentially used to manufacture wearable antimicrobial hemostatic fabrics 231. Luo *et al.* co-doped GO and Pt nanoparticles into a metal-organic framework (MOF, NH_2_-MIL-125), and this complex presents a promising photothermal effect under conditions of white light irradiation. The complex can be used to develop effective bactericides. It was shown that the antibacterial efficiency of the complexes against *S. aureus* and *E. coli* was as high as 99.94 and 99.12%, respectively. The results were achieved within 20 min of white light irradiation 232. Wang *et al.* prepared a composite hybrid material containing Zn nanosheets and biodegradable MOF that can effectively kill bacteria and promote wound healing in bacterial infections 233. Hybrid nanosheets of Zn and GO exhibit excellent antibacterial properties and accelerate the process of wound healing 234. The modification of MOFs with metal ions and poly(dopamine) imparted excellent antimicrobial properties to the materials 235. Metal-based MOF particles can be further polymerized with quaternary ammonium chitosan to synthesize photosensitive hydrogels, which exhibit excellent photothermal properties when irradiated with near-infrared light. The materials inhibit bacterial metabolism and respiration by charge transfer, resulting in bacterial death 236. This hybrid material can be used to effectively treat bacterially infected open wounds in pathogenic bacteria-contaminated environments.

Composite materials can effectively integrate the advantages of individual forms. These materials exhibit antibacterial properties, boast of accelerated wound healing ability, and are characterized by shape memory properties. These materials are the focus of current research and are used to develop hemostatic materials.

### Medical applications

Hemostatic materials are applied in various medical fields based on their properties, and currently, the wound healing and antimicrobial characteristics of these materials are the focus of research. Corneal healing and bone repair require the assistance of hemostatic materials. In addition, hemostatic materials can effectively reduce the degree of dissemination of tumor cells caused by bleeding. These materials are being extensively researched to develop improved materials with surgical applications.

#### Wound healing

Uncontrollable bleeding is frequently associated with patient death. Various hemostatic materials have been used to develop commercial formulations that can be used to realize hemostasis in humans. For example, Hemcon^®^ (based on chitosan), TraumaDEX^®^ (potato starch-based microparticles), and QuickClot^®^ (based on zeolite), *etc.* are examples of such materials. However, the existing commercial materials exhibit poor biodegradability, cannot be used to treat complex wounds, and are difficult to remove. Hence, researchers are constantly trying to develop improved hemostatic materials.

**Figure 6:**
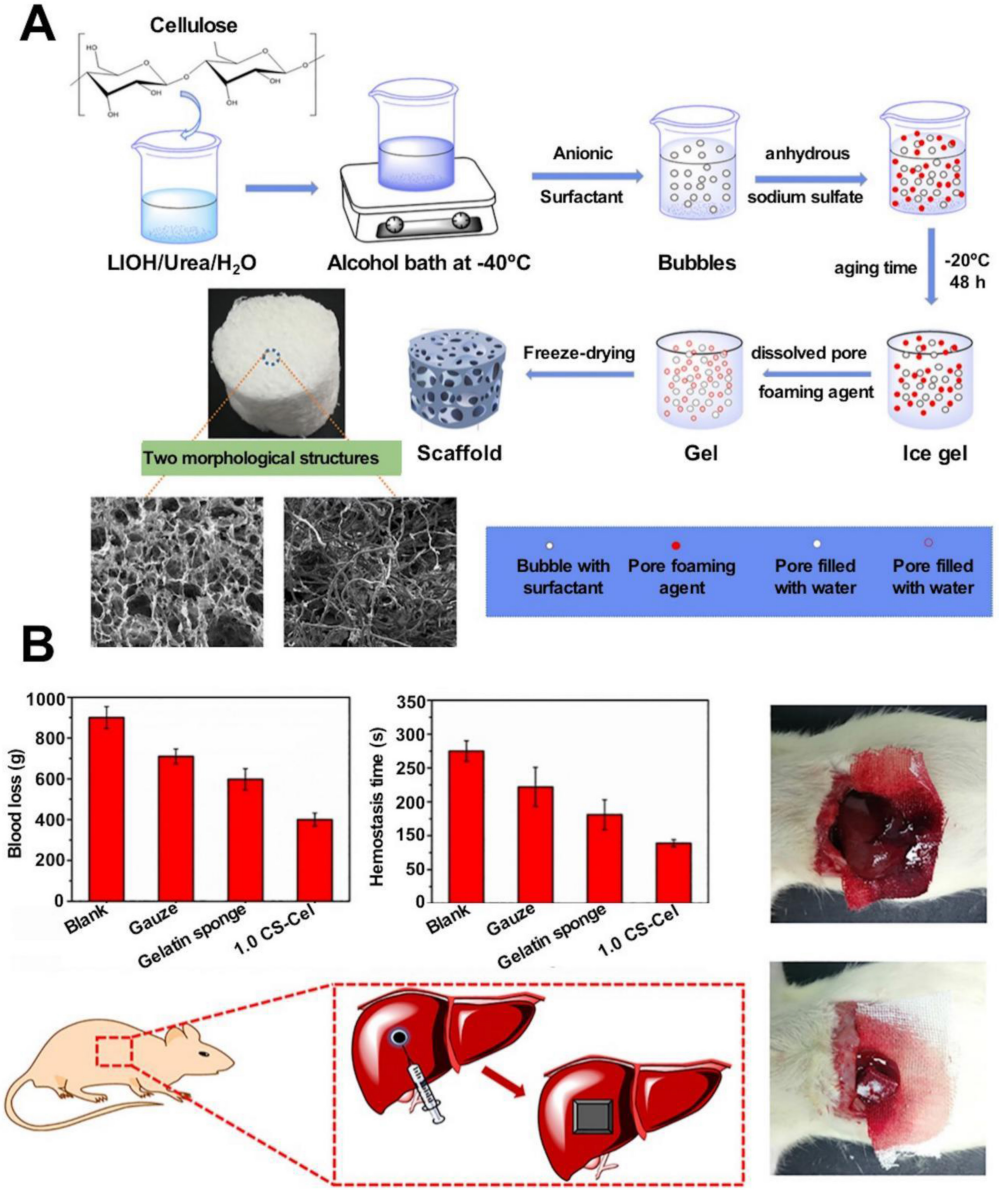
Cellulose/chitosan sponges are available for deep wound hemostasis. A: schematic diagram of the process of preparing cellulose sponges with porogenic agents and surfactants; B: evaluation of the hemostatic effect of composite sponges applied to a rat liver trauma model. Reproduced with permission from 23. ©2020 Elsevier Ltd. All rights reserved.

Natural polymers are biocompatible and degradable. Chitosan, a positively charged natural polysaccharide, exhibits excellent hemostatic and antibacterial properties. Hence, it is being widely researched as a hemostatic material. Chitosan-based hemostatic materials exist in the forms of nanofibers, hydrogels, and nanoparticles. Composite materials can also be formed using chitosan. The diameters of the chitosan-based nanofibers prepared following the electrostatic spinning method are in the range of 9.2±3.7 nm. Thus, the materials boast of a high surface area that is in contact with wounds. These exhibit excellent hemostatic effects in hepatic perforation models in rats, rabbits, and pigs 237. The chitosan-based hydrogel can moisten wounds to promote healing and stop bleeding. These hydrogels exhibit certain antibacterial properties to prevent wound infection 238. Biranje *et al.* assembled chitosan nanoparticles into a chitosan dressing under conditions of lyophilization. They used it to study an *in vitro* model of human skin fibroblasts, and the results revealed that the sample could be used to accelerate the process of thrombin formation and effectively promote wound healing 239. Composite materials integrate the advantages of multiple components. A composite sponge was prepared from cellulose and chitosan. Cellulose imparted good water absorption and rapid shape recovery properties, and chitosan imparted good antibacterial properties to the composite sponge. Fan *et al.* used composite sponges to study the mouse severed tail model, the rat liver injury model, and the rat femoral artery trauma model. The properties of the prepared sponge were compared with the properties of the gelatin sponges. It was observed that the composite sponges significantly shortened clotting time and reduced bleeding 23 (Figure 6).

Synthetic polymers are stable systems that can be easily prepared in large quantities and functionalized. Most synthetic polymers are not bioactive and thus cannot promote blood clotting. These cannot be considered as substrates or cross-linking agents for hemostatic materials. Hemostatic materials based on synthetic polymers are prepared as nanofibers, hydrogels, nanoparticles, and sponges. PCL nanofibers exhibit excellent biocompatibility and degradability and are FDA-approved medical materials that are being used in the field of hemostasis research. PCL nanofibers loaded with carmine can effectively accelerate the process of wound healing. These reduce scar tissue areas in diabetic mice wound models 240. Self-assembling peptides can be used to treat various types of wounds. The short peptide RADA16 can rapidly self-assemble into nanofibers in an ion-rich environment, and the nanofiber barrier can rapidly stop bleeding when the material is used locally in the brain, liver, and femoral artery hemorrhage models. The peptide breaks down into amino acids and can further promote wound healing at the damaged site 241. The injectable hydrogels based on polyglutamic acid and tetra PEG can be gelated rapidly *in situ*. These materials exhibit high mechanical strength and can be used to close large arterial bleeding sites to significantly shorten the hemostasis time and reduce blood loss 242. PVA sponge is characterized by excellent water absorption ability. These can be used for effective hemostasis, and the materials function through expansion-local physical compression cycles 132.

The first inorganic material to be used clinically to control bleeding was zeolite. QuickClot^®^ was carried by U.S. troops in 2002. Zeolites can rapidly absorb fluid from the blood. These help to concentrate local clotting factors and platelets and promote the formation of blood clots. However, heat is released during the process, which can potentially damage tissues 243. Liang *et al.* cross-linked zeolite with graphene sponges and controlled the process of heat release by exploiting the heat conduction ability of graphene to eliminate the thermal effect of zeolite. The process helped to control the wound temperature below 42 °C effectively. Rapid hemostasis was observed in the rat arterial injury model, and the use of the material shortened the bleeding time significantly. The bleeding time was shorter than the bleeding time recorded when the control group was treated with Quikclot^®^ 155. CNF can promote fibrin growth and blood clot formation, and its hydrophobic surface reduces the possibility of secondary bleeding when the dressing is removed. The deposition of CNfs on the surface of a gauze or sponge can significantly increase its surface area 244.

Metal ions such as silver, zinc, and copper exhibit excellent broad-spectrum antibacterial properties, heat resistance, and dispersibility. Metal ions are commonly used to prepare composite hemostatic materials. These are combined with other hemostatic active ingredients to prepare hemostatic materials. It is difficult to heal wounds in diabetic patients, and these wounds are associated with a high incidence of bacterial infections. Chronic nerve and vascular damage are also observed in diabetic patients. Nosheen Masood *et al.* blended silver nanoparticles with antimicrobial properties with a solution consisting of PEG and chitosan to form a hydrogel in the presence of glutaraldehyde (a cross-linking agent). They used the material to treat a diabetic rabbit wound model as the material exerted antioxidant and antimicrobial effects and promoted wound healing 245.

#### Prevention of tumor recurrence

Hemostatic materials are regularly used in surgical procedures. Surgery is the preferred and most effective way to treat common solid tumors (those associated with lung, breast, and colorectal cancers). Tumor tissues have an abundant local blood supply and contain multiple abnormal neovascularizations. Thus, bleeding inevitably occurs during surgery, posing difficulties during surgical procedures. However, it adversely affects disease prognosis 246. Intraoperative bleeding significantly increases the risk of tumor recurrence attributable to the metastasis of cancer cells 247. Although some commercial hemostatic materials such as QuickClot^®^, glutaraldehyde-cross-linked albumin 248, and fibrin bandages 249 are used to treat superficial bleeding conditions, simple gauze tamponade is primarily used during surgery in the cases of malignant tumors. This significantly increases surgical risks. Rapid hemostasis should be achieved using hemostatic materials. Therefore, the hemostatic materials should be able to improve patient prognosis and prevent the recurrence of postoperative tumors.

**Figure 7:**
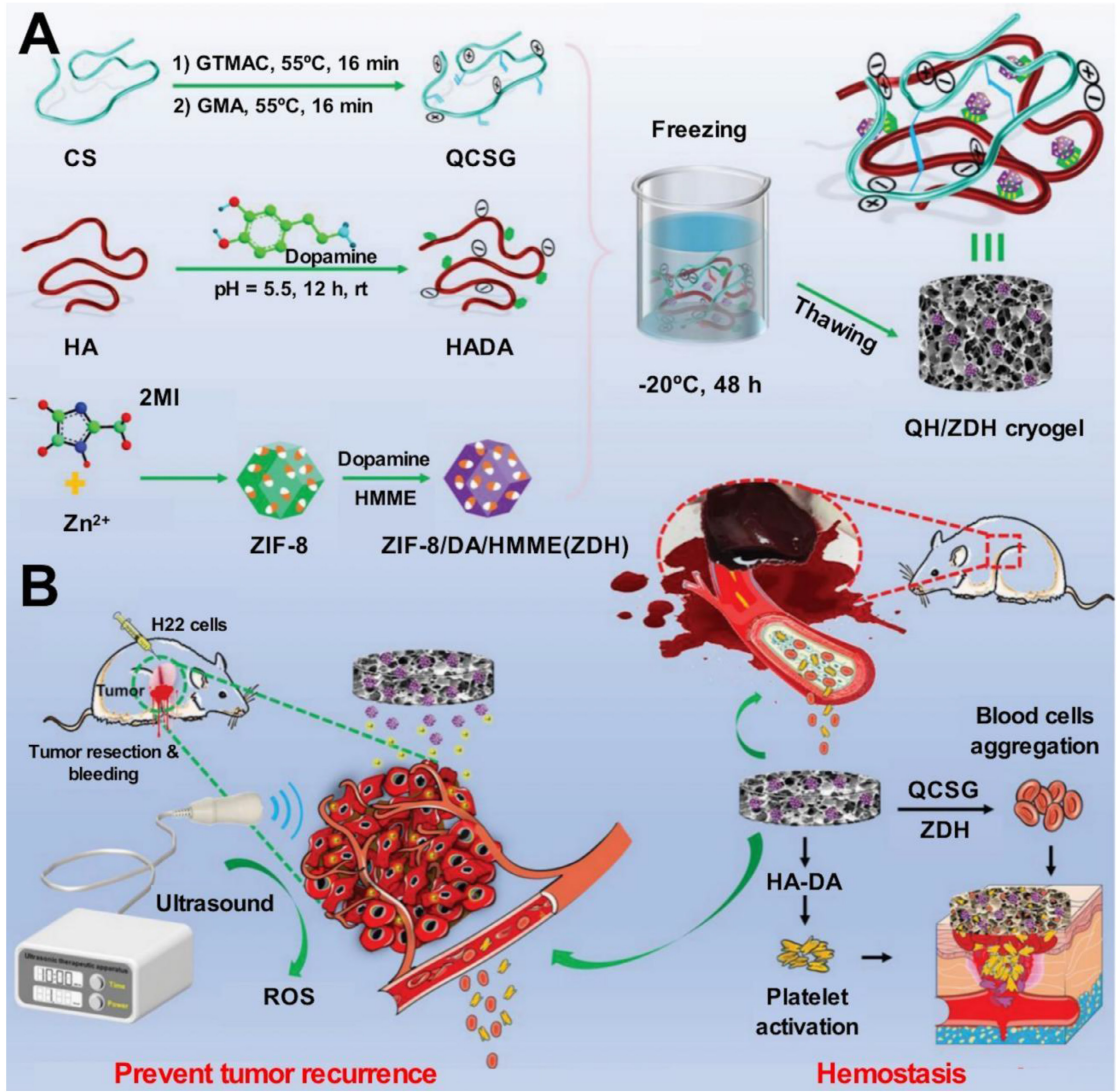
Schematic diagram of the synthesis of cryogels and their application in the prevention of tumor recurrence. A: schematic diagram of the synthesis of QCSG/HA-DA/ZDH composite cryogel; B: the role of composite cryogel in hemostasis and prevention of tumor recurrence. Reproduced with permission from 251. © 2021 Wiley‐VCH GmbH. QCSG: glycidyl methacrylate-functionalized quaternized chitosan; HADA: dopamine-modified hyaluronic acid; HMME: hematoporphyrin monomethyl ether; ZDH: ZIF-8/DA/HMME.

It is important to develop advanced integrated therapeutic solutions (hemostatic materials) that can rapidly stop bleeding and load anti-tumor drugs to advance the field of oncology treatment. In addition to exerting hemostatic effects, hemostatic gelatin sponges loaded with 5-FU can release drugs continuously and target local tumors. These materials exhibit significantly high degrees of anti-tumor effects when used to treat (*in situ*) a mouse colorectal cancer model 250. The applicability of gelatin sponges is limited by the low strength and rapid degradation rate of the materials. These materials exert a limited effect on extensively bleeding surgical sites. Hence, various composites have been developed. Guo* et al.* combined the advantages of multiple hemostatic activities to prepare zeolite nanoparticle-enhanced multi-network cryogels. Materials based on quaternized chitosan, dopamine-modified HA, and ultrasound-sensitizer-loaded dopamine-modified ZIF-8 were developed, and the cross-linking between the components was realized at -20 ℃ following the cryogelation technique. The crystalloid network exhibits excellent water absorption performance, effectively concentrates local blood cells and platelets, and improves the clotting effect. These properties can be attributed to the high-density through-pore structure of the material. Rapid and efficient hemostasis could be achieved when it was used to address large-volume defects associated with the liver. Reactive oxygen species (ROS) were produced under the action of ultrasound, and acoustodynamic anti-cancer effects were recorded. It was used to study the mouse ectopic liver tumor model, and it was observed that it could significantly improve the survival rate of mice after surgery 251 (Figure 7). Combinations of sponges and nanofibers could also be used to achieve intraoperative hemostasis and generate antitumor effects. Chen *et al.* used alginate and gelatin sponges cross-linked following the interpenetrating polymer network (IPN) strategy to exert hemostatic synergy. The system was combined with the curcumin-loaded PCL-PEG-PCL nanofibers to control the accumulation of curcumin at the tumor surgical sites. The experiments were conducted using a mouse subcutaneous tumor recurrence model. The results revealed that the material could significantly inhibit tumor recurrence 252. The chitosan/gelatin sponge prepared following the freeze-drying method was characterized by the presence of a sandwich-like layer of adriamycin-regalactone fibers in the middle. The sponge could safely and efficiently implant the composite material locally into the surgical resection cavity to prevent bleeding at the surgical site. These could release drugs to inhibit tumor recurrence. Postoperative patient mortality could be reduced, and patient prognosis could be improved 253.

The appropriate selection and application of hemostatic materials can effectively reduce intraoperative bleeding. Hemostatic materials can help clear the surgical field clear and reduce the difficulty of surgery. The materials can also be used to reduce tumor metastasis attributable to blood. In addition, the extent of postoperative recurrence can be reduced, and the prognosis can be improved. Thus, developing effective hemostatic materials with a clinical application is important.

#### Bone repair

Bone defects are a common complication of trauma, inflammation, infection, and bone tumor resection. The bleeding caused by bone defects can be attributed to the injury of cancellous bone, which is loose in structure and rich in blood flow. In most cases, blood oozes out from cancellous bones, and hemostasis by vasoconstriction is difficult. It is difficult to stop bleeding using hemostatic gauze or following conventional methods such as electrocoagulation, clamping, and collagen sponge filling 254. Liu* et al.* assembled quaternized branched-chain starch and tannic acid onto the surface of porous starch to construct multilayer microspheres to treat hemorrhage in cancellous bone defects. The composite microspheres consisting of branched starch and tannic acid could promote platelet adhesion, activation, and aggregation of RBCs, exhibiting optimal hemostatic properties. Inadequate inflammatory response and pro-bone repair properties were observed when the material was used to study a mouse cancellous bone defect model. Further proof-of-concept models in Beagle dogs confirmed the positive effect of composite microspheres, and it was observed that the composites could be used to treat bleeding bone defects in large mammals 255. Loading calcium phosphate and chitosan layers on the surface of electrospun gelatin could provide rapid hemostasis. Application of the material in a rat skull defect model revealed that it could significantly promote the process of bone regeneration 256.

Severe infections accompany bone defects resulting from trauma. Wang *et al.* prepared a bone defect-filling material with a sequential release system that rapidly releases hydroxypropyltrimethyl ammonium chloride chitosan, a potent antimicrobial agent, to kill potentially infected bacteria. The bone morphogenetic protein-2 (BMP2) is gradually released, and the process of bone repair is promoted 257.

**Figure 8:**
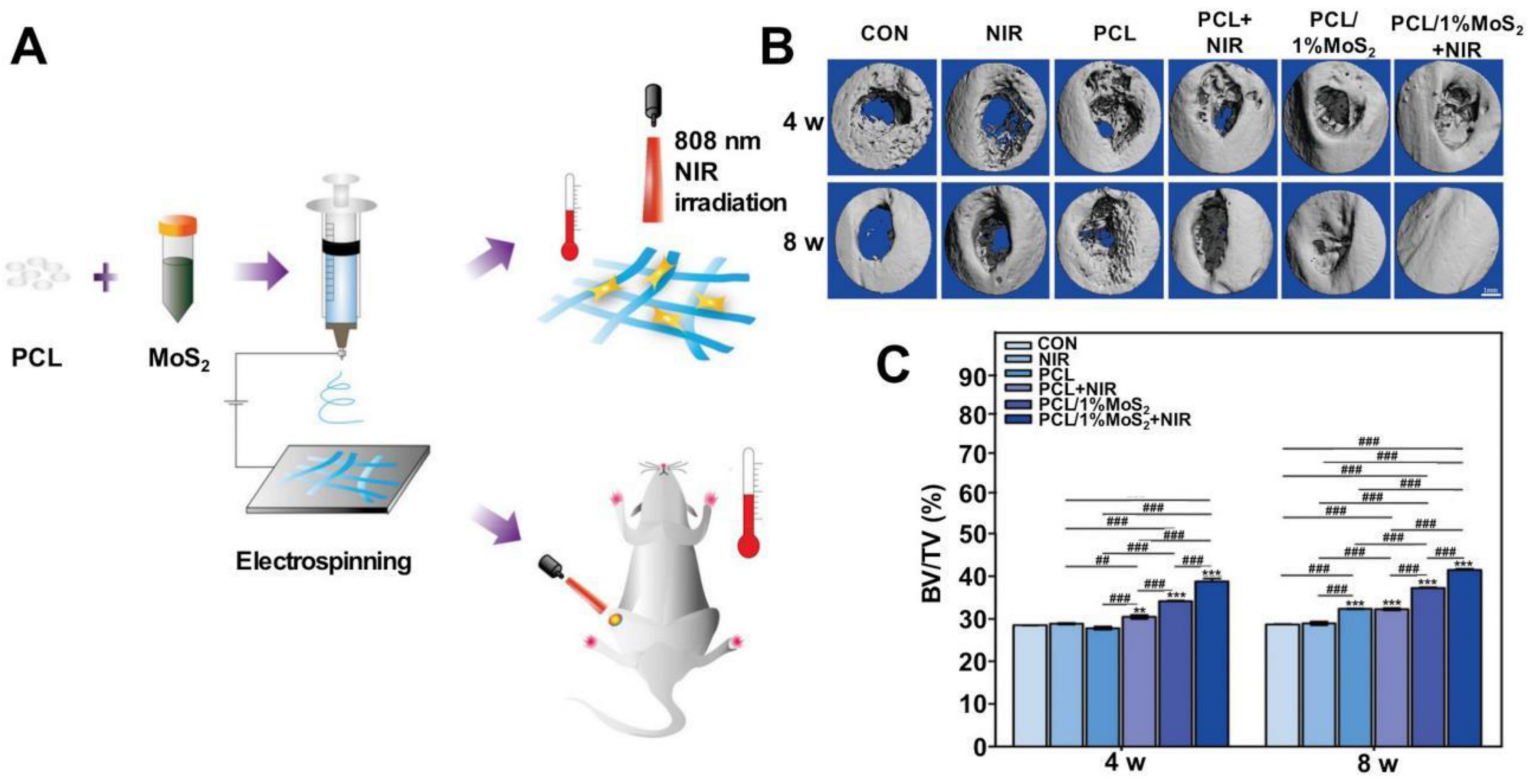
Electrospun PCL/MoS_2_ nanofiber membranes could accelerate bone regeneration. A: schematic diagram of PCL/MoS_2_ nanofibrous membrane preparation and promotion of osteogenesis *in vivo*; B: microCT images and bone volume/total volume (BV/TV) ratio analysis after implantation of PCL and PCL/1% MoS_2_ in a rat tibial defect model. Reproduced with permission from 258. © 2021 Wiley-VCH GmbH. PCL: poly(ε-caprolactone)

A combination of PCL nanofibers and molybdenum disulfide significantly promotes the growth of bone mesenchymal stem cells *in vitro*. The composite could accelerate osteogenesis and the process of bone healing when applied to a rat tibial bone defect model 258 (Figure 8). The introduction of polyacrylic acid into PVA-based hydrogels can significantly enhance the mechanical strength of the material and improve the cell adhesion property. It can be used as a cartilage tissue replacement that can maintain the cell growth morphology and promote the processes of bone regeneration and repair 196. Hydroxyapatite, alumina ceramics, calcium carbonate, and other inorganic materials are also used as scaffolds in the field of bone tissue engineering 259. In addition to promoting bone repair, some hemostatic materials with antimicrobial properties can be used to treat osteomyelitis. For example, magnetic composites prepared from molybdenum disulfide and iron oxide have been used to treat bacterially infected osteomyelitis effectively 260.

#### Eye diseases

**Figure 9:**
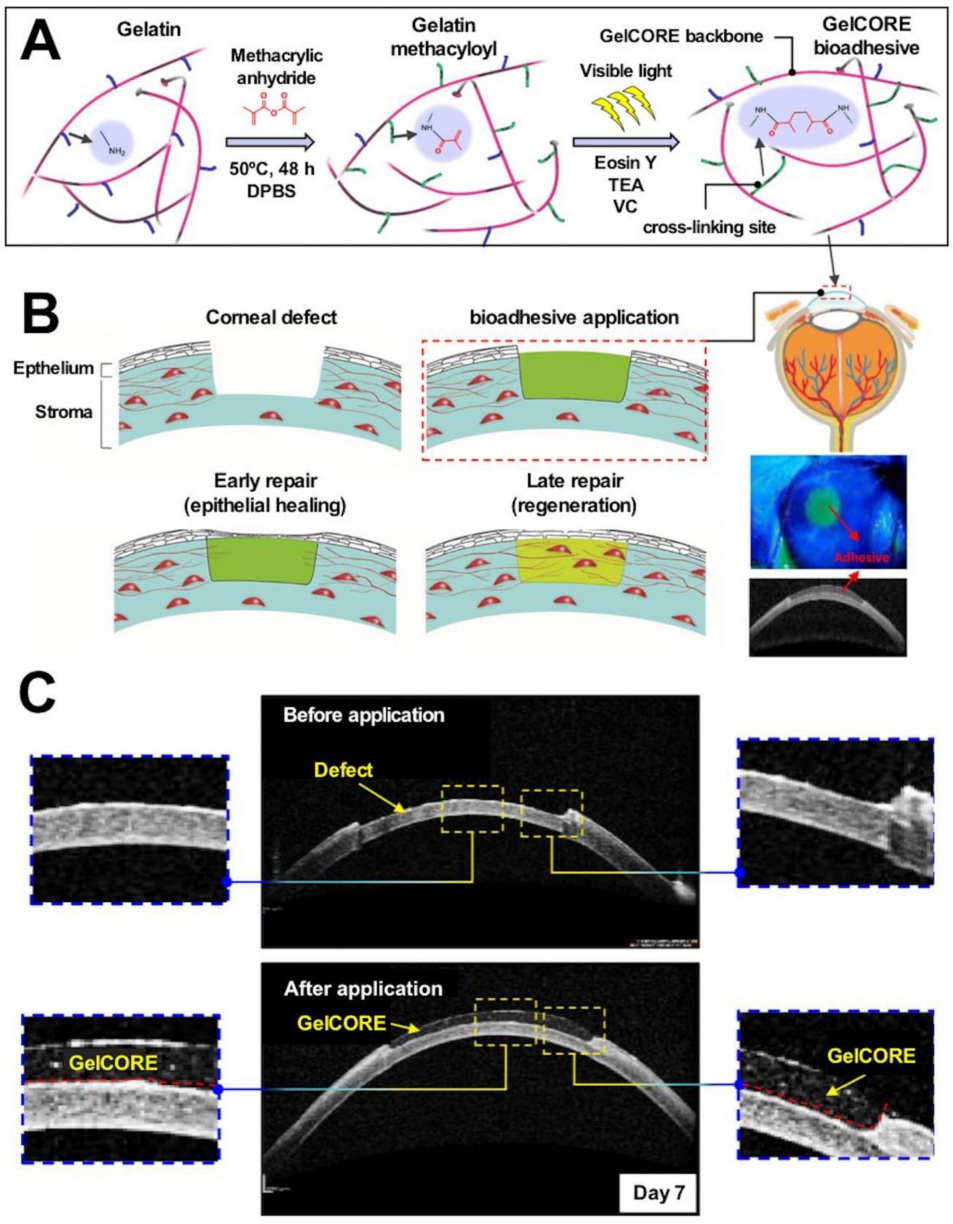
Gelatin-based naturally adherent hydrogels (GelCORE) can be a suitable tool for repairing corneal damage. A: schematic diagram of GelCORE synthesis; B: schematic diagram of GelCORE for rapid and long-term repair of corneal damage; anterior segment optical coherence tomography images of a rabbit corneal defect model C before and after application of GelCORE. Reproduced with permission from 264. ©2019 Authors.

Hemostatic materials should be carefully chosen to treat special areas of the body. The transparent cornea is the first line of defense of the eye against external forces. It forms the foundation of the visual function. Corneal damage is a common cause of visual impairment. The usage of biomaterials for corneal repair can effectively reduce patient costs as the process is cheaper than the transplantation process, which suffers from a severe donor shortage 261, 262. The selection of hemostatic materials for the treatment of corneal injury should take into account the following relevant properties: 1) excellent biocompatibility and biodegradability; 2) high transparency; 3) mechanical stability and support; 4) high adhesion to tissue; 5) ability to promote endogenous tissue regeneration; and 6) stable physicochemical properties and ease of application. Multiple natural and synthetic polymers have been developed as tissue adhesives for corneal sealing and repair. The natural polymer chitosan exhibits excellent mucosal adhesion and antimicrobial properties. Moreover, it promotes corneal wound healing. Therapeutic drugs can be incorporated into chitosan gels to protect them from degradation and improve the extent of absorption 263. Sani *et al.* designed a gelatin-based adhesive consisting of gelatin and a photo-initiator that can be photo-crosslinked following a short exposure to visible light to form a hydrogel that adheres firmly to the corneal surface. The researchers used the adhesive to study a rabbit corneal stromal wound model and demonstrated that it could effectively seal corneal defects and induce stromal regeneration. The combination of gelatin and photoinitiator is facile to apply. The composite significantly improves the contact time of the adhesive with the cornea. This helps reduce the frequency of administration and improves patient compliance 264 (Figure 9). Collagen, a natural polymer, is the equivalent of type I collagenous protofibrils present in human connective tissues. It is biocompatible and permeable and promotes the processes of corneal epithelial and stromal regeneration 265. Chae* et al.* used collagen vitrigel to study a rabbit corneal stromal wound model and revealed that collagen vitrigel effectively promoted corneal repair. The material could be used to maintain the original tissue transparency. It could also be used to reduce the degree of inflammatory responses generated and inhibit neovascularization 266. Natural polymers are biocompatible. However, the degree of mechanical stability is low. ReSure Sealant^®^, a PEG-based adhesive, was the first to be approved by the FDA in 2014 for the treatment of corneal incisions following IOL placement for cataract surgery 267. Shehata *et al.* tested the ability of ReSure^®^ to withstand elevated intraocular pressure in a rabbit corneal wound model 268. It was observed that ReSure^®^ could not adhere strongly to ocular surfaces, and it could easily get detached from the surfaces in wet ocular environments. It also could not promote the processes of corneal epithelial and stromal regeneration. Various composite materials are being prepared for application in the field of managing corneal wounds. Chitosan-based thermosensitive *in situ* prepared hydrogels remain in the liquid state at room temperature. They get converted to the gel state as the hydrogen bonds break with an increase in temperature. Gratieri *et al.* prepared (*in situ*) a hydrogel based on PEO-PPO-PEO and chitosan. The experimental results revealed that 50-60% of the hydrogel remained in contact with the corneal surface after 10 min of dropping them into the human eye. This significantly increased the retention time 269. The addition of chitosan nanoparticles to chitosan/polycaprolactone gels can effectively improve the mechanical properties and antibacterial ability of the composites. This also helped maintain the corneal shape and surface-wetting properties. Matrix regeneration was also promoted 270.

#### Tooth Repair

Tooth extraction is a common surgical procedure. Oral and maxillofacial surgeries are performed to extract incurable affected teeth and pathogenic teeth that cause other diseases. Surgeries are also performed to extract teeth to introduce orthodontics or prosthetics. A surgeon chooses an appropriate hemostatic material to fill the bleeding wound to prevent secondary bleeding or bacterial infection. The oral cavity is the second largest microbial habitat in the body after the intestinal tract. Thus, with its antimicrobial properties, chitosan can be effectively used for oral hemostasis. Chang *et al.* prepared a composite hydrogel from chitosan and polyglutamic acid and used it to treat the Wistar rat incisor extraction model in a controlled group. The effects exerted by gelatin sponges, pure chitosan, and composite hydrogels were compared. The results indicated that the composite hydrogel could effectively promote the process of formation of new bones in the alveolar sockets after tooth extraction. The use of hydrogel could reduce the occurrence of infection 271. Chitosan exhibits anti-bacterial and anti-fungal properties and the application of chitosan hydrogel can effectively reduce pain and inflammation following dental procedures 272. Fibrin hydrogels can also be used for hemostasis after tooth extraction. Sarkar *et al.* conducted a clinical randomized controlled trial that included 60 patients with extracted teeth. The patients were subjected to conditions of oral antiplatelet therapy and randomized to apply either fibrin-based hydrogels or chitosan-based hydrogels for hemostasis. The trial revealed that chitosan hydrogels were more effective than fibrin-based hydrogels for hemostasis. These significantly reduced clotting time. However, the fibrin-based hydrogels were better at promoting the process of wound healing 273.

**Figure 10:**
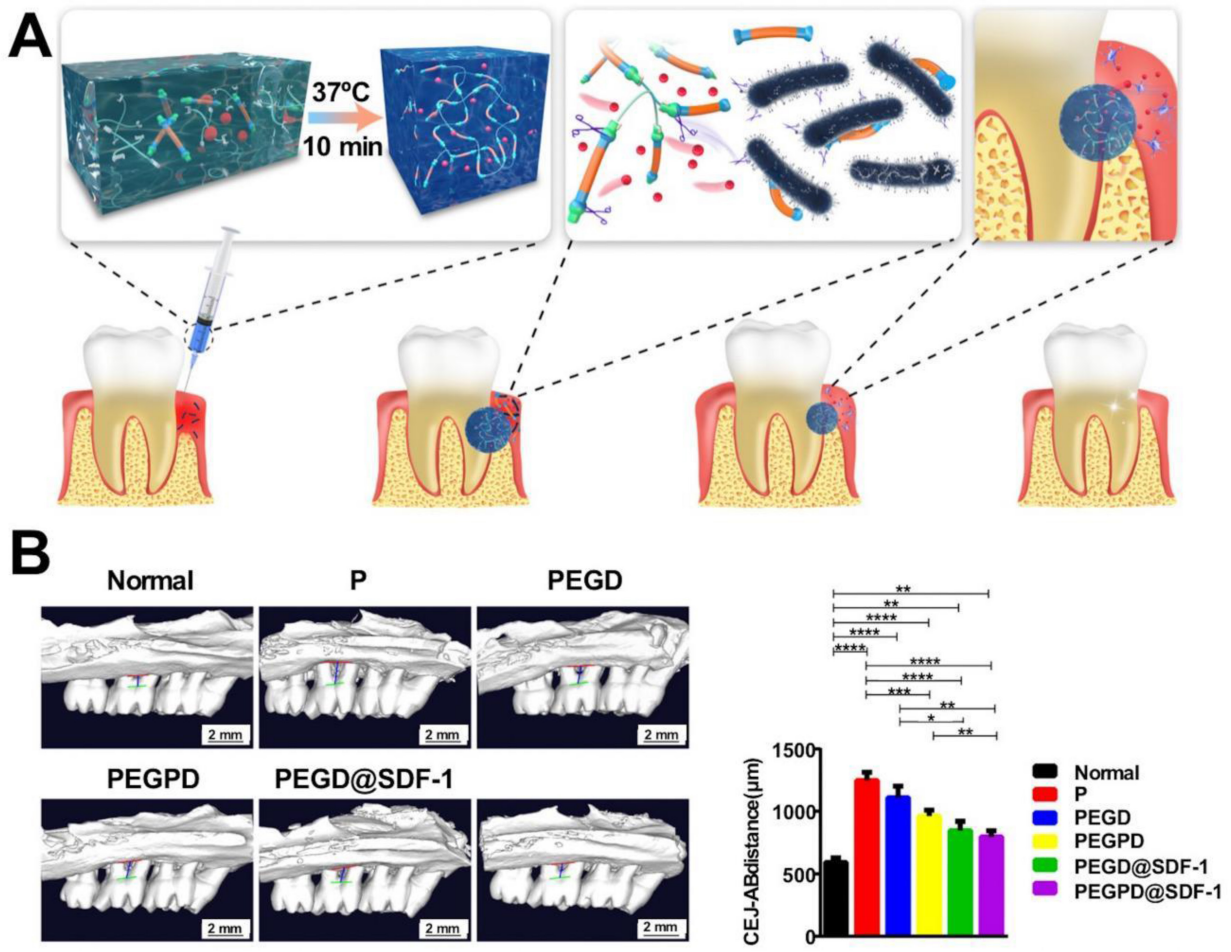
PEG-DA-based thermosensitive hydrogels are available for therapeutic use in periodontal disease. A: schematic diagram of the synthesis of *in situ* thermosensitive hydrogel; B: micro-CT analysis of the effect of *in situ* hydrogel injection on periodontal bone regeneration after one week. Reproduced with permission from 276. ©2021 American Chemical Society. PEGD: polyethylene glycol diacrylate; SDF-1: stromal cell derived factor-1

Periodontal disease is a common oral disease and is one of the leading causes of tooth loss in adults. It is primarily manifested by inflammation and bleeding of gums, resorption of alveolar bone, loosening and displacement of teeth, and tooth loss 274. Chitosan promotes tissue healing and induces alveolar bone formation. It plays a vital role in guided tissue regeneration techniques 275. Liu *et al.* prepared a thermosensitive responsive hydrogel loaded with stromal cell-derived factor-1 (SDF-1) and used it to study a rat periodontitis model. The results revealed that the material could inhibit inflammation and promote the regeneration of alveolar bone 276 (Figure 10). Zhang* et al.* prepared a sandwich-like composite scaffold consisting of chitosan/PCL/gelatin. The scaffold was biocompatible and mechanically stable. It swelled and adhered well to cells. Sufficient oxygen and energy for the cell and tissue regeneration processes were obtained via the pores 100.

### Conclusion and Perspectives

In this review, we have discussed the advantages and disadvantages of using different components or materials used to develop hemostatic materials. Three aspects have been primarily considered: active hemostatic ingredients, material forms, and medical applications. Natural polymers, synthetic polymers, inorganic materials, and materials containing metals are the four main categories of materials used to realize hemostasis. Chitosan-based nanofibers, hemostatic sponges, and hydrogels have been used to obtain satisfactory results in animal models characterized by low bleeding volumes. However, excellent performance could not be achieved for the cases of high-bleeding wounds. Collagen, gelatin, and other natural polysaccharides can potentially cause allergic reactions. Synthetic polymers are readily available and can be conveniently and functionally modified. The poor bioactivity of synthetic polymers, which can be produced in bulk, hinders their practical application. Inorganic materials have shown long-term physical and chemical stability without bio-toxicity. Zeolite emits a lot of heat when it encounters water or blood. This results in an increase in the local temperature of the wound. This can potentially cause skin burns and hinder the process of wound healing. The various metal ions used to prepare the hemostatic materials impart excellent antibacterial properties to the materials. The metal ion also improves the coagulation efficiency of the materials. There is a growing trend to design smart hemostatic materials that can mimic natural hemostatic processes. For example, platelet mimics that promote thrombus contraction and fibrin-structured nanofibers that facilitate clot formation may point toward the development of novel hemostatic materials.

At present, most commercially available hemostatic materials are designed for large, flat wounds, and materials to treat deep, irregular wounds should be developed. Flexible hemostatic materials that adhere tightly to the surrounding tissue should be developed to treat wounds near joints to avoid dressing dislodgement. Excessive bleeding hinders adhesion. Hemostatic materials prepared in the form of hydrogels and sponges adhere well to tissues. Methods to safely remove or degrade the materials following use should be researched. Hemostatic materials should be removed safely without causing pain and secondary bleeding (due to exfoliation). For degradable materials, the relation between the rate of degradation and the pace of hemostasis and tissue repair needs to be considered. The degradation products should not induce inflammatory responses or obstruct blood vessels *in vivo.* An ideal hemostatic material should have the following characteristics: a) it should have the ability to be used directly at bleeding sites, and the hemostatic time should be short; b) it should be inexpensive and convenient to apply; c) it should have the potential to be mass-produced, there should not be differences in the properties of the materials produced in different batches; d) it should be stable and portable and should be characterized by a prolonged storage time; e) it should be absorbable, should not require cleaning, and should not cause tissue damage or infection.

## Figures and Tables

**Table 1:** Various components and forms of hemostatic materials used to realize hemostasis.

Material	Form	Testing model	Efficacy	Ref.
Chitosan	Chitosan/PVA/ZnO nanofiber mat	Diabetic rabbit subcutaneous wound model	Excellent antibacterial properties observed; promotes the production of granulation tissues and wound healing at the injured tissue.	18
	Chitosan/silk fibroin/ Mg (OH)_2_ nanoparticles hydrogel	*In vitro*	The composite material exhibits favorable biocompatibility and can effectively inhibit the growth of *Pseudomonas aeruginosa*.	19
	O-nitrobenzyl alcohol/ carboxymethyl chitosan hydrogel	Rat liver injury model	Exhibits great adhesion and sealing properties; hemostasis is achieved within a short time period; decreases bleeding volume.	20
	Chitosan/Diatom-Biosilica Aerogel	Rat hemorrhage model.	Exhibits excellent water absorption properties; promotes the aggregation of red blood cells and adhesion and activation of platelets; accelerates hemostasis.	21
	Alkylated chitosan/ diatom-silica sponge	Rat tail transect model	Exhibits excellent biocompatibility; induces the activation, deformation, and aggregation of red blood cells and platelets, thus activating the intrinsic coagulation pathway and rapidly stopping bleeding.	14
	Quaternized chitosan/CNT cryogel	Mouse-liver injury model and mouse-tail amputation model	Demonstrates strong mechanical strength, superior blood absorption rate, and remarkably high blood cell and platelet adhesion and activation capacity.	22
	Cellulose/chitosan sponge	Mouse tail amputation model	Exhibits excellent absorbent and rapid shape recovery properties; exhibits effective antibacterial properties; cellulose/chitosan sponges have been studied *in vitro* and *in vivo* to achieve rapid hemostasis.	23
HA	GelMA/HA-NB hydrogel	Porcine carotid artery hemorrhage and heart injury model	Gels quickly and adheres tightly to seal bleeding arteries and heart walls; withstands blood pressure of up to 290 mmHg, making it a superior wound sealant.	24
Gelatin	Gelatin nanofiber sponge	Rabbit ear artery injury model and liver trauma model	The composite sponge is characterized by low density, high surface area, and excellent fluid absorption capacity; aggregates and activates platelets in large quantities; accelerates the formation of platelet emboli; activates the extrinsic and intrinsic coagulation pathways.	25
	Gelatin sponge with thrombin	Pig liver bleeding model	Easy to apply and can stop bleeding quickly to reduce the amount of blood loss realized.	15
Peptide	Self-assembling peptide RADA16 nanofiber	Mouse diabetic wound model	Accelerates wound closure, collagen deposition, and tissue remodeling in healthy and diabetic mice.	26
	Polylysine/thiolated chitosan hydrogel	Rat liver injury model	Gels quickly and exhibits excellent tissue adhesion properties.	27
PEG	Tetra-PEG hydrogel	Rabbit liver hemorrhage model	Exhibits rapid gelation properties, strong tissue adhesion ability, high mechanical strength, rapid degradation properties, and controlled dissolution properties.	28
	TRAP-loaded starch/PEG sponge	Rat femoral artery and liver hemorrhage models	The superior water absorption property of the sponge helps absorb plasma, concentrate blood cells, and enhance blood clotting. Post water absorption, the shape-fixed sponge exhibits sufficient mechanical strength to apply pressure to the wounds to promote hemostasis.	29
PU	PU nanoparticle	Rat neurosurgical model	Hemostatic agents containing PU NPs exhibit anti-inflammatory and neuroprotective property *in vivo*; promotes procedures involving fragile tissues or organs.	30
	PU based adhesive	Porcine vascular suture model	Applied to porcine vascular sutures to effectively prevent perioperative suture line bleeding; does not produce an inflammatory response that affects healing.	31
PVA	PVA/oxidized betaine polysaccharide Schiff base aerogel	Whole skin defect model	Exhibits promising antimicrobial and hemostatic properties that help reduce inflammation; promotes angiogenesis; accelerates epithelialization to accelerate wound healing.	32
	PVF sponge with pore-filled chitosan hydrogel, CaCO_3_ particle, and silica particle	Wistar rat liver hemorrhage model	The application of PVF-based composites significantly reduced the bleeding time (78.3-90.4%) compared to the commercial hemostatic product Celox™.	33
PCL	PCL/gelatin nanofibers loaded with 1% lawsone	Rat wound model	PCL/Gel nanofibers loaded with 1% lawsone significantly improves the cell attachment and proliferation properties; promotes wound healing.	34
	CNT-coated PCL nanofiber	Rat calvarium bone defect animal model	Inhibits inflammatory response; promotes angiogenesis; drives MSC adhesion; promotes bone regeneration.	35
PLGA	PLGA/PEG/ SiO_2_ nanofiber	Porcine liver laceration model	The composite nanofibers can be deposited directly onto the surgical site in the form of a solid fiber pad for ease of application. It exhibits excellent biocompatibility and sealing properties.	36
PEO	PEO/chitosan nanofiber mat	Porcine liver laceration model	Model experimental studies have confirmed the superior biocompatibility and hemostatic properties of PEO/chitosan nanofiber mats, which can be used to repair liver damage and achieve a high biodegradation rate.	37
Silica	Silica/chitosan sponges	Mouse tail amputation model	The silica/chitosan sponges are characterized by promising water absorption properties and mechanical strength; these help achieve excellent hemostasis in the mouse tail amputation model.	14
	Silica nanoparticles for loading tannic acid	Mouse tail amputation model	Superior antibacterial properties and hemostatic properties.	38
	Chitosan/hydrocaffeic acid/SNP	Rat femoral artery trauma model	It can form a network structure with fibrin in the blood to improve the mechanical strength of blood clots and promote hemostasis and wound healing ability.	39
Graphene	GO/gelatin aerogel	*In vitro*	Exhibits good water absorption properties; negative charge on its surface can promote hemostasis; red blood cells adhere to its surface, facilitating the formation of a stable fibrin network that promotes hemostasis.	40
	Graphene/MMT composite sponge	Rabbit artery injury model	The composite sponge exhibits good water absorption capacity and can be used to rapidly stop bleeding in a rabbit arterial injury model without remaining in the blood vessels and causing thrombosis.	41
	CNF/copper-containing glass aerogel	*In vitro*	CNF/copper-containing glass aerogel can promote angiogenesis effectively; exerts excellent bactericidal effects, which promote hemostasis and wound healing.	42
Platelet-derived nanovesicle	Natural platelet-derived nanoparticles prepared by hypotonic ultrasonication	Mouse tail transection bleeding model	The natural, biocompatible platelet-derived nanoparticles can be used to achieve hemostasis when injected; inflammatory reactions are not caused.	43
Platelet mimic	Liposomes with surface-decorated collagen and vWF-binding peptide	Mouse tail transection bleeding model	The surface of the liposomes is modified using a variety of mimetic peptides, which can effectively mimic the processes of platelet adhesion and aggregation to form platelet thrombi and achieve hemostasis.	44

(PVA: polyvinyl alcohol; CNT: carbon nanotube; HA: hyaluronic acid; GelMA: gelatin methacrylate; PEG: polyethylene glycol; TRAP: thrombin receptor agonist peptides; PU: polyurethane; PVA: polyvinyl alcohol; PVF: poly(vinyl formal); PCL: polycaprolactone; PLGA: poly(lactic-co-glycolic acid); PEO: poly (ethylene oxide); SNP: silicon dioxide-based nanoparticle; GO: graphene oxide; CNF: carbon nanofiber)

## Abbreviations

HA: hyaluronic acid
PEG: polyethylene glycol
PU: polyurethane
PVA: polyvinyl alcohol
PCL: polycaprolactone
PEO: poly (ethylene oxide)
EDTA: ethylenediaminetetraacetic acid
OC: oxidized cellulose
TEMPO: 2,2,6,6-tetramethyl-1-piperidinyloxy
DTP: 3,3'-dithiopropionic acid
HA-DTPH: mercapto-hyaluronic acid derivative
HA-tyr: HA-tyrosine
HA-Ph: phenolic hydroxy HA
AOS: alginate oligomers
PEGDA: poly(ethylene glycol)diacrylate
FDA: Food and Drug Administration
PMMA: polymethylmethacrylate
PPO: poly(propylene oxide)
GO: graphene oxide
GMCS: graphene/montmorillonite composite sponges
MMT: montmorillonite
ALP: alkaline phosphatase
PLLA: poly(lactic acid)
FMP: fibrinogen mimetic peptide
PLGA-PLL: poly(lactic-co-glycolic acid)-poly-L-lysine
CNT: carbon nanotube
SGH: self-assembled graphene hydrogel
EDC: 1-ethyl-3-(3-dimethylaminopropyl) carbodiimide
GelMA: gelatin methacrylate
HAMA: methacrylated HA
CMC: carboxymethyl chitosan
SA: sodium alginate
TA: tannic acid
MFC: microprotofibrillated cellulose
cCNCs: carboxylated cellulose nanocrystals
SCD: supercritical drying
SD: spray drying
OD: oven drying
PVF: poly(vinyl formal)
TRAP: thrombin receptor agonist peptides
LNPs: liposome-based nanoparticles
SNPs: silicon dioxide-based nanoparticles
ROS: reactive oxygen species
IPN: interpenetrating polymer network
BMP2: bone morphogenetic protein-2
SDF-1: stromal cell-derived factor-1
